# Pasta, a Versatile Transcriptomic Clock, Maps the Chemical and Genetic Determinants of Aging and Rejuvenation

**DOI:** 10.1002/advs.76740

**Published:** 2026-07-27

**Authors:** Jérôme Salignon, Maria Tsiokou, Patricia Marqués, Enriqueta Rodríguez‐Diaz, Hazel Ang, Federico Pietrocola, Christian G. Riedel

**Affiliations:** ^1^ Department of Medicine Huddinge Karolinska Institute Huddinge Sweden; ^2^ Department of Molecular Biosciences The Wenner‐Gren Institute Stockholm University Stockholm Sweden; ^3^ Department of Cell and Molecular Biology Karolinska Institute Solna Sweden

**Keywords:** bioinformatics, biology, cellular aging, computational biology, microarray, senescence, transcriptome

## Abstract

With the growing burden of age‐related diseases, understanding and modulating the aging process has become a priority. Transcriptomic aging clocks (TACs) can track biological age but remain limited by platform dependence, tissue specificity, or restricted accessibility. To address this, we developed Pasta, a robust and broadly applicable human TAC, built using a novel ‘age‐shift’ learning framework. Pasta accurately predicted relative age across diverse tissues and data types, including bulk and single‐cell RNA‐Seq as well as microarray data. Its predictions aligned with senescent and stem‐like cellular states and relied on model coefficients enriched for p53 and DNA damage response pathways. Pasta's age scores correlated with tumor grade and patient survival in several cancer types, indicating potential clinical relevance. Applied to over three million transcriptomes from the Connectivity Map L1000 dataset, Pasta identified both established and previously unrecognized age‐modulatory compounds and genetic perturbations, highlighting mitochondrial translation and mRNA splicing as key determinants of cellular propensity for aging and rejuvenation, respectively. Experimental validation confirmed pralatrexate as a potent senescence inducer and piperlongumine as a rejuvenating agent in human cells. Together, these findings establish Pasta as a versatile and accessible tool for aging research and therapeutic discovery.

## Graphical abstract

Pasta is a transcriptomic aging clock built on an age‐shift learning framework and trained on 17 000 samples across 21 datasets. It accurately predicts relative biological age across tissues, platforms, and species, captures stemness‐to‐senescence transitions, and identifies age‐modulatory perturbations. Applied to over three million transcriptomes, Pasta reveals mitochondrial translation and mRNA splicing as key determinants of cellular aging and rejuvenation.

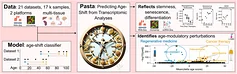

## 1 Introduction

The growing prevalence of chronic non‐communicable diseases in later life has positioned aging as a major driver of pathology. This, coupled with advances in understanding the fundamentals of aging and health [1, 2], has intensified interest in pharmacological and lifestyle strategies to modulate aging [3]. Unlike chronological aging, which progresses at a constant pace, biological aging is modulated by endogenous and environmental factors that can accelerate, decelerate, or even reverse aging processes [4]. This highlights biological age as a latent but measurable trait that reflects current and future health, captures individual variability in aging rates, and offers a means to evaluate geroprotective interventions.

Over the past decade, a range of computational tools—collectively termed ‘aging clocks’—have been developed to quantify biological age and estimate aging rates. These models draw from diverse molecular and clinical hallmarks across multiple ‘omics’ layers, including epigenetic, transcriptomic, metabolomic, proteomic, and metagenomic data [5]. Among them, epigenetic clocks based on genome‐wide CpG methylation patterns have been widely used due to their high precision in estimating chronological and biological age, strong predictive value for morbidity and mortality, and the relative stability of such DNA methylation (DNAm) marks, which enable the capture of cumulative aging effects [6]. However, DNAm clocks also have limitations, including reduced sensitivity to rapid or transient changes [7] and limited biological interpretability, as CpGs are not always clearly linked to gene function [8]. In addition, single‐cell DNAm approaches remain costly, technically demanding, and lack standardized protocols, restricting their utility in resolving aging at single‐cell resolution [6].

Transcriptomic aging clocks (TACs) address these limitations: they sense transient shifts, yield interpretable gene and pathway outputs, and can be applied to extensive bulk [9], single‐cell [10, 11], spatial [10, 12], genetic [13, 14], and chemical [13, 15] perturbation datasets. Notably, ongoing efforts include constantly growing transcriptomic perturbation resources, such as the Connectivity Map (CMAP) L1000 [13], Perturb‐Seq datasets [14], or the Tahoe‐100 dataset [15]. Applying TACs to such datasets could reveal regulators of aging and rejuvenation with translational potential. For example, compounds that raise the biological age of cancer cells may enhance therapeutic efficacy [16], and gene perturbations that accelerate aging in iPSC‐derived neurons can improve models of late‐onset disease [17]. Conversely, agents that lower age in normal cells may promote regeneration; improving stem cell fitness [18], iPSC protocols [19], or partial reprogramming cocktails [20]. While a handful of age‐modulatory perturbations have recently been described [21], systematic approaches to identify them remain limited. A recent study showed the potential of such an approach by mining the Gene Expression Omnibus (GEO) [22] database using aging clocks to find novel age‐modulatory perturbations [23]. Another landmark and untapped resource in this context is the CMAP L1000 dataset [13], which contains over three million transcriptomes from 248 cell lines exposed to more than 14 000 gene perturbations and 30 000 compounds. Identifying both general and cell line‐specific age‐modulatory perturbations in this dataset could inform the development of more effective age‐related translational strategies.

Human TACs have been developed in blood [24], muscle [25], fibroblasts [26], skin [27], and multi‐tissue settings [28, 29, 30, 31, 32, 33], and more recently at single‐cell resolution using blood [34], immune cells [35, 36], and PBMCs [37]. Despite this progress, no TAC has achieved widespread adoption comparable to epigenetic clocks, due to limitations in performance, availability, and platform compatibility. Gene expression sensitivity to environmental cues enables detection of transient states but also introduces variability and noise, requiring robust modeling across heterogeneous datasets. Most human TACs, including single‐tissue models and the multi‐tissue RNAAgeCalc [29], are trained on single datasets, leading to overfitting and limited generalization. Consistently, MultiTIMER [30], trained on multiple datasets, outperforms RNAAgeCalc [29] despite being trained on fewer samples (∼3000 vs ∼9600). Another key barrier is availability: DeepQA [32], and Shokhirev & Johnson ’s [28] TACs offer no usable software, while RAPToR [31] requires users to generate custom references without default human datasets. Moreover, most multi‐tissue TACs [28, 29, 30, 32] require raw data reprocessing, batch correction, or model retraining, limiting their use to bulk RNA‐Seq data and reducing reproducibility. While the multi‐species tAge [33] clock circumvents these issues, it was trained on only four human tissues, leaving it unclear how well it performs in other human tissues.

To address these limitations, we present Pasta (Predicting Age‐Shift from Transcriptomic Analysis), a ready‐to‐use transcriptomic aging clock (TAC) applicable across multiple tissues and experimental platforms. Pasta showed improved performance over MultiTIMER [30] and tAge [33]—the current leading multi‐tissue human TACs—across diverse datasets, generated biologically meaningful age estimates in mouse tissues profiled using different platforms, and revealed a substantial contribution of p53‐related genes to its output. Beyond age prediction, Pasta effectively distinguished between senescent, quiescent, and stem cells, in both static and dynamic settings, indicating its capacity to capture bidirectional cell state transitions from stemness to senescence. Clinically, we observed that the Pasta age score could stratify tumor grade and survival for several cancers, which may reflect its ability to detect senescence‐ and stemness‐related features in tumors. When applied to the CMAP L1000 dataset [13], Pasta identified potential age modulators such as pralatrexate and piperlongumine, which we experimentally validated, and it pinpointed molecular features associated with cell‐line responsiveness to pro‐ or anti‐aging interventions. Taken together, Pasta offers a robust and interpretable framework for biological age estimation across tissues, platforms, and species, and for systematic discovery of genetic or chemical modulators of aging and rejuvenation, with potential relevance to translational research, including in oncology [16], neurodegeneration [17], and regenerative medicine [18, 19, 20].

## 2 Results

### 2.1 Construction of a General‐Purpose Transcriptomic Aging Clock From Heterogeneous Multi‐Tissue Data

Given that existing human TACs often show limited transferability, being trained on single cohorts or requiring extensive preprocessing, we aimed to develop a broadly applicable transcriptomic aging clock that can be used across tissues and sequencing platforms. To ensure broad generalization, we compiled heterogeneous transcriptomic data from the Genotype‐Tissue Expression (GTEx) [38] project, the Gene Expression Omnibus (GEO) [22], and the Expression Atlas [39], retaining 17 212 healthy samples spanning multiple tissues from 21 studies (18 bulk RNA‐Seq and 3 microarray; Figure 1a, Table S1). Gene expression values were rank‐transformed to mitigate platform and processing effects.

**FIGURE 1:**
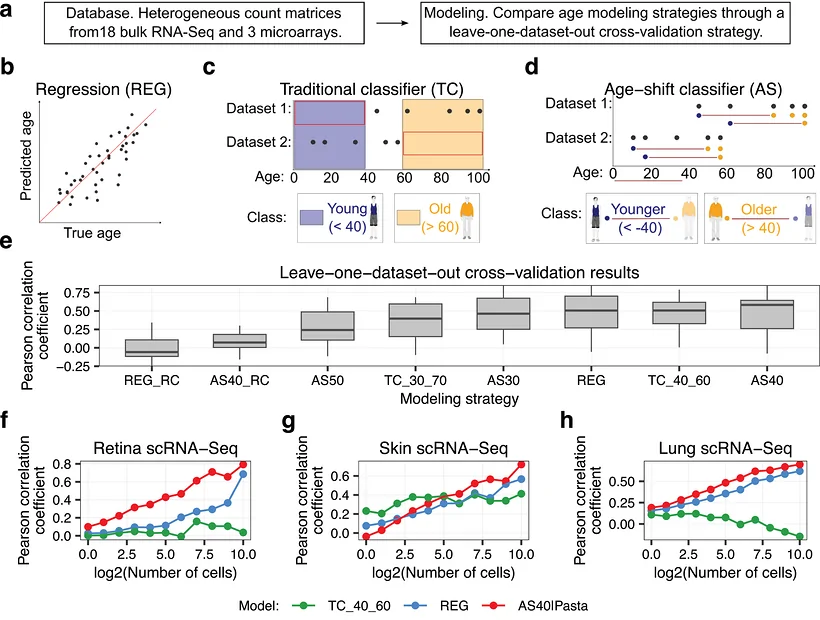
The age‐shift classifier enables accurate cross‐platform prediction of relative transcriptomic age. (a) Overview of the evaluated modelling strategies. Models were trained and tested exclusively on samples from healthy donors. (b–d) Schematic diagrams of the regression (b), traditional classifier (c), and age‐shift classifier (d) strategies. (e) Comparative performance in predicting relative age, assessed using leave‐one‐dataset‐out cross‐validation. All samples were rank transformed except for REG_RC and AS40_RC where raw counts were used. The y‐axis shows the Pearson Correlation Coefficient (PCC) between chronological ages and predicted age scores in the held‐out datasets. (f–h) Age predictions for single‐cell RNA‐Seq aging atlases of the retina [45] (f), skin [46] (g), and lung [44] (h). Abbreviations: REG, regression; AS30/40/50, age‐shift classifier with 30, 40, or 50 year age difference; TC_30_70 and TC_40_60, traditional classifiers with young cutoffs at 30 or 40 years and old cutoffs at 70 or 60 years; RC, raw counts.

Using this dataset, we compared three modeling strategies: regression models predicting chronological age (Figure 1b), young‐versus‐old classifiers (Figure 1c), and age‐shift classifiers trained on sample pairs separated by a defined age gap (Figure 1d)—the latter being an approach conceptually similar to twin neural network regression [40, 41] and pairwise difference regression [42] or classification [43]. For the classifiers, predictions were converted to age scores (see Methods).

We evaluated all models using a leave‐one‐dataset‐out (LODO) strategy. An age shift model with a 40‐year age gap achieved the highest median Pearson correlation coefficient (PCC) across all samples (Figure 1e) and remained the top performer even when restricting the evaluation to young and old individuals only (Extended Data Figure S1a). It also showed consistent performance across tissues and platforms (Extended Data Figures S1b, S2, and S3). Models trained on unranked counts performed poorly (median PCC < 0.1), underscoring the importance of rank transformation (Figure 1e; Extended Data Figure S1a).

To assess generalization to data types unseen during training, we tested our top models on single‐cell RNA‐Seq datasets. Because training relied on bulk data, single‐cell profiles were aggregated into pseudobulks of increasing size (1 to 1024 cells) to evaluate performance at different resolutions. Across retina, skin, and lung datasets, the age‐shift model consistently outperformed the regression model and the young‐versus‐old classifier (Figure 1f–h) [44, 45, 46]. Notably, the latter failed when test datasets lacked age categories present during training (Figure 1f,h), highlighting the limitation of fixed age cutoffs.

Altogether, the 40‐year age‐shift model provided the most robust and generalizable predictions of relative age across heterogeneous transcriptomic data, displaying superior performance over previous approaches. We refer to this model as Pasta throughout the remainder of this study.

### 2.2 Pasta Leverages p53‐Related Genes to Accurately Predict Relative Age Across Platforms, Tissues, and Species

To benchmark Pasta, we compared it with MultiTIMER [30] and tAge clocks [33], the most accurate available multi‐tissue human TACs. Pasta showed the highest predictive performance in 6 of 8 datasets from our LODO analysis (Figure 2a; Table S2) and in 4 of 6 independent validation datasets (Figure 2b). These datasets encompassed diverse tissues (Table S2), confirming Pasta's broad applicability across tissue types. Since Pasta works across tissues and platforms, we wondered if it could also work across species. We extended Pasta for usage in mouse by leveraging one‐to‐one orthologues (see Methods). Encouragingly, we observed that Pasta could also predict age accurately in mouse across a variety of tissues and platforms (Figure 2c,d). Altogether these results show that Pasta is an accurate predictor of relative age across tissues, platforms, and species.

**FIGURE 2:**
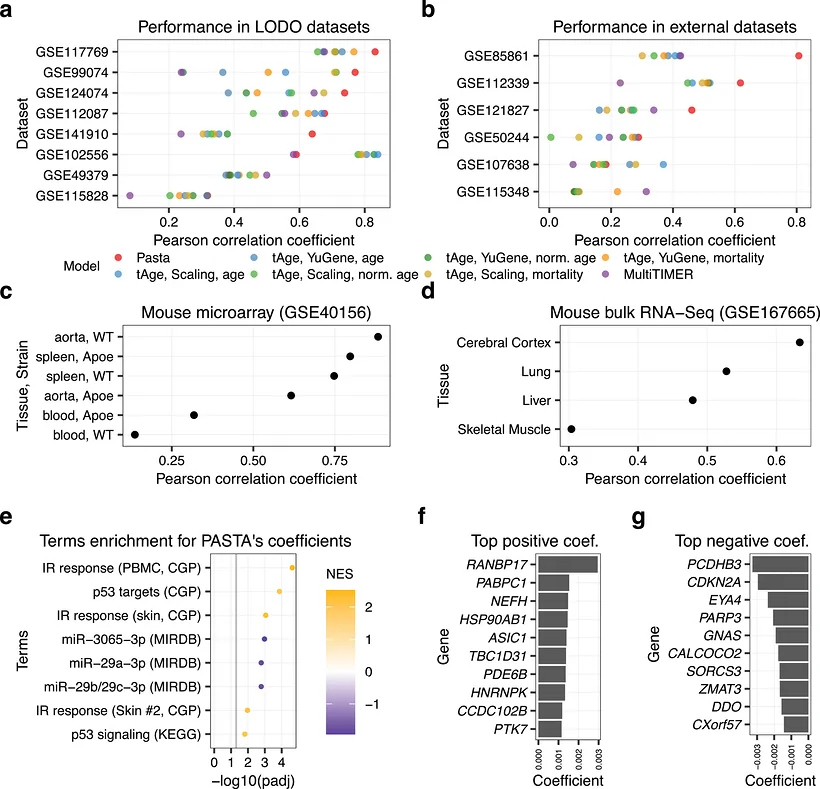
Pasta predicts transcriptomic age across tissues through p53‐linked signals. (a, b) Pearson Correlation Coefficient (PCC) of age scores for Pasta, MultiTIMER [30], and elastic net multi‐species tAge clocks [33], compared with chronological ages for healthy individuals. (a) Results from the leave‐one‐dataset‐out analysis. (b) Results on external validation datasets. (c, d) PCC of age scores for Pasta compared with chronological age for mouse datasets GSE40156 (c) and GSE167665 (d). (e) Gene set enrichment analysis (GSEA) of Pasta coefficients using the REACTOME, CGP, KEGG and miRDB gene sets from the Human MSigDB Collections. The gray lines mark the adjusted p‐value cutoff of 0.05. (f, g) Top ten genes with the most positive (f) and negative (g) coefficients.

To assess reproducibility of Pasta's predictions, we used the SEQC/MAQC‐III benchmark dataset [47], comprising four reference RNA samples sequenced at 6 independent laboratories with 4 library replicates per sample per site. 98.8% of variance in Pasta age scores was attributable to sample identity (Extended Data Figure S4a), with between‐site Coefficient of Variation (CV = 4.05%) comparable to within‐site CV (2.91%), confirming that inter‐laboratory variation was small relative to biological signal. Furthermore, age scores increased monotonically with known mixing fractions (PCC = 0.936, Extended Data Figure S4b), indicating sensitivity to gradual age score changes.

We next conducted gene set enrichment analysis (GSEA) on broakdly used collections (REACTOME, miRDB…) [48] using Pasta's coefficients. Genes withpositive coefficients, whose higher expression predicted older age, were enriched for p53 signaling and irradiation response terms, pointing to activation of the DNA damage‐p53 axis [49] (Figure 2e; Table S3). The second and third strongest negative enrichments, genes whose higher expression predicted younger age, were targets of miR29, a p53 activator [50] which drives aging phenotypes in mice [51]. Notably, no pathway was significantly enriched when using the baseline regression model generated in Figure 1, suggesting that the age‐shift learning framework more effectively captures aging‐relevant transcriptional signal.

Consistent patterns were observed at the individual gene level, with *RANBP17* carrying the largest negative coefficient, nearly twice that of any other gene (Figure 2f). This gene promotes cell proliferation [52] and has been proposed to be a master regulator of aging, which decreases with age [53]. The strongest positive coefficients belonged to *PCDHB3*, a colorectal‐cancer tumour‐suppressor [54], *CDKN2A* (p16), a canonical p53 target and senescence marker, and *ZMAT3*, another key p53 target [55] (Figure 2g).

In conclusion, Pasta is a robust multi‐tissue and multi‐platform transcriptomic age model that grounds its predictions in tumor‐suppressor genes and core aging modulators.

### 2.3 Pasta Accurately Tracks Cellular Age Across Senescence, Pluripotency, and Differentiation States

Given Pasta's strong performance across tissues, we next asked whether it could also track cellular aging in vitro, focusing on senescent and stem cells, which represent opposite ends of the cellular age spectrum. Here, cellular age refers to transcriptomic state rather than elapsed time, with stem cells occupying a transcriptionally plastic state with high proliferative potential and senescent cells representing a relatively stable state characterized by durable growth arrest and accumulated stress.

Pasta accurately distinguished senescent from proliferative cells in 19 out of 30 RNA‐Seq datasets (AUC‐ROC = 1) and achieved moderate discrimination (AUC‐ROC > 0.7) in five additional datasets (Figure 3a; Table S4, median delta age score of 31.3), consistently labeling senescent cells as older. It also distinguished senescent from quiescent cells in 4 of 5 datasets (Figure 3b, median delta age score of 33.5). In a 450‐day serial passaging experiment of primary fibroblasts that reached replicative senescence [56], Pasta's age scores increased almost linearly with culture duration (PCC = 0.896; Figure 3c). We next assessed whether Pasta could detect chemical inducers of senescence in single‐cell data. Combining two studies that tested the same 138 compounds in A549 cells, one using scRNA‐Seq [57] and another using flow cytometry with a senescence reporter [58], Pasta assigned significantly higher age scores to senescence‐inducing compounds than to other compounds (*p* = 9.9 × 10^−6^; Welch's two‐sample t‐test; Figure 3d).

**FIGURE 3:**
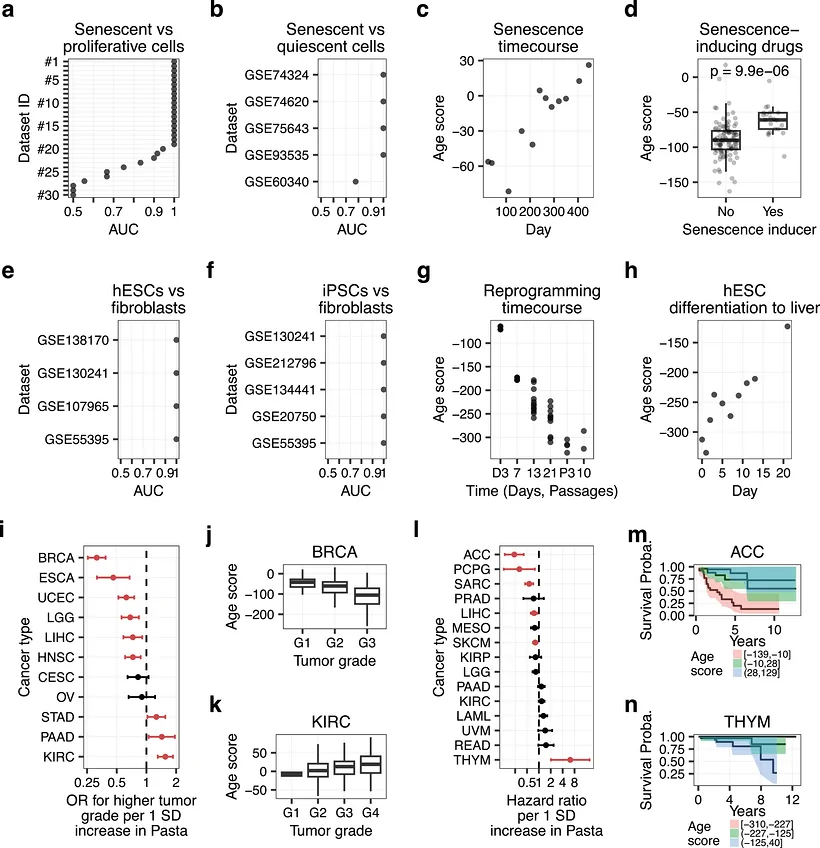
Pasta identifies senescent and stem cells and may help stratify tumor grade and patient overall survival. (a‐b) Pasta's ability to discriminate between senescent and proliferative (a) or quiescent (b) cells, assessed using AUC‐ROC (Area Under the Curve of the Receiver Operating Characteristic) values, across multiple RNA‐Seq datasets. (c) Pasta's predictions on a microarray senescence time course dataset [56]. (d) Pasta was applied to pseudobulks from a SciPlex dataset generated by exposing the A549 cell line to 138 drugs [57], and the resulting age scores were evaluated for their capacity to recover senescence‐inducing drugs, as reported in Wang et al. 2017 [58]. Only the highest drug concentration (10 µM) was used for this analysis. (e, f) Pasta's ability to discriminate between stem cells (e) or induced‐pluripotent stem cells (iPSCs) (f) and fibroblasts, assessed by AUC‐ROC. (g) Pasta's age scores on a bulk RNA‐Seq OSKM‐mediated reprogramming time‐course dataset [59]. (h) Pasta's age scores on a bulk RNA‐Seq liver differentiation time‐course dataset [60]. (i) Forest plot of odds ratios for higher tumor grade per 1 standard deviation increase in Pasta's age score. Points show cancer‐specific estimates with 95% confidence intervals on a logarithmic scale. Red and black indicate significant and non‐significant false discovery rate–adjusted likelihood ratio test results, respectively. (j, k) Boxplots of Pasta's age scores for breast invasive carcinoma (j) and kidney renal clear cell carcinoma (k) patients, grouped by tumor grade. (l) Forest plot of hazard ratios per 1 standard deviation increase in Pasta's age score. Points show cancer‐specific estimates with 95% confidence intervals on a logarithmic scale. Cancer types are restricted to the 15 with the largest absolute deviation of the hazard ratio from 1 for visualization. Red and black indicate significant and non‐significant false discovery rate–adjusted likelihood ratio test results, respectively. (m–n) Kaplan–Meier survival estimates for adrenocortical carcinoma (m) and thymoma (n) patients, grouped by age score tertiles.

Having established that Pasta can detect senescent and thus very old cells, we next tested its sensitivity to very young cell states. Pasta effectively distinguished embryonic stem cells from fibroblasts across four bulk RNA‐Seq datasets (Figure 3e, median delta age score of −381) and induced pluripotent stem cells from fibroblasts in five datasets (Figure 3f, median delta age score of −256), consistently labeling stem cells as younger. During OSKM‐mediated fibroblast reprogramming [59], Pasta's age scores declined linearly with time (PCC = −0.905; Figure 3g), whereas in directed differentiation of human embryonic stem cells toward liver cells [60], age scores increased steadily (PCC = 0.926; Figure 3h).

Together, these results show that Pasta reliably tracks not only tissue age but also cellular age across a continuum spanning pluripotent, differentiated, and senescent states.

### 2.4 Pasta Reveals Cancer Type‐Specific Associations Between Age Score, Tumor Grade, and Patient Survival

Studies applying transcriptomic stemness models to The Cancer Genome Atlas (TCGA) transcriptomes have shown that higher stemness correlates with advanced tumor grade and poorer survival in cancer patients [61, 62]. Recently, increased cancer cell age has also been linked to adverse outcomes [16], as the accumulation of senescent cells within tumors, irrespective of whether they are normal or cancerous, can promote inflammation, relapse, and immune evasion [16, 63, 64, 65]. Yet, TCGA transcriptomes have not been systematically examined using a senescence prediction model or an aging clock, leaving it unclear whether poor prognosis reflects stemness alone or also senescence within bulk tumor tissue. Because Pasta's age score captures the balance between stem‐like and senescent‐like programs, we applied Pasta to TCGA tumor transcriptomes to quantify these states.

Multivariate proportional odds models adjusted for chronological age and demographic and clinical covariates showed that the association between Pasta's age score and tumor grade depended on the cancer type. In six of eleven cancers, adding the age score improved model fit and was associated with lower odds of high‐grade disease, whereas in three cancers it improved model fit in the opposite direction, corresponding to higher odds of high‐grade tumors. In two cancers the age score did not improve model fit (Figure 3i–k, Table S5).

In survival analyses, Cox models with the same covariate adjustments showed that adding the age score improved prognostic performance in five of 31 tumor types and was associated with reduced mortality; and in one cancer, thymoma, it improved model fit in the opposite direction, corresponding to increased mortality, while the remaining cancers showed no improvement in model fit (Figure 3l–n, Table S6).

Taken together, the age score was associated with tumor grade and survival in several cancer types, with both protective and adverse relationships observed, consistent with contributions from senescence and stemness related programs to cancer progression and outcome. Inclusion of the age score improved model fit beyond chronological age and covariates, supporting Pasta's utility as a measure of biological age.

### 2.5 Systematic Identification of Age‐Increasing and Rejuvenating Small Molecules in the CMAP L1000 Dataset

Having established Pasta's predictive power for tissue and cellular aging in health and disease, we next assessed whether it can also identify age‐modulatory perturbations.

To this end, we applied Pasta to the CMAP L1000 dataset [13], which contained the transcriptomic responses of 248 cell lines to more than 30 000 chemical and 14 000 genetic perturbations, totaling more than three million transcriptomes. Coincidentally, the bead‐based nature of the L1000 assay provided also an opportunity to test Pasta's cross‐platform robustness. Delta age scores were calculated for each sample on each plate by subtracting negative control age scores (DMSO for compounds, untreated or empty vectors for genetic perturbations) from perturbation age scores. Then, mean and significance of delta age scores across cell lines were computed for each perturbation.

By this approach, we identified 271 compounds that were significantly associated with higher delta age scores and 63 compounds with lower delta age scores, hereafter termed *Aging* and *Rejuvenating* compounds, respectively (Figure 4a; Table S7). As expected, top Aging compounds included canonical senescence inducers such as mitoxantrone, gemcitabine, and doxorubicin [66], whereas top rejuvenating compounds included pluripotency‐promoting molecules, such as RepSox and PD0325901 [67]. Annotating the CMAP library with known senescence‐ [66] and reprogramming‐ [67] related drug classes revealed that 18.1% of Aging compounds belonged to senescence‐inducing classes, compared with less than 0.2% among all remaining compounds (*p* = 6.23 × 10^−75^, Fisher's exact test, Figure 4b). Similarly, 15.9% of Rejuvenating compounds belonged to reprogramming‐related classes, compared with less than 0.2% among all remaining compounds (*p* = 6.12 × 10^−18^, Fisher's exact test). Notably, 27% of Rejuvenating compounds were annotated as both pro‐senescence and pro‐reprogramming, all of which were histone deacetylases (HDAC) inhibitors, a drug class known to induce both reprogramming [68, 69] and senescence [70, 71, 72].

**FIGURE 4:**
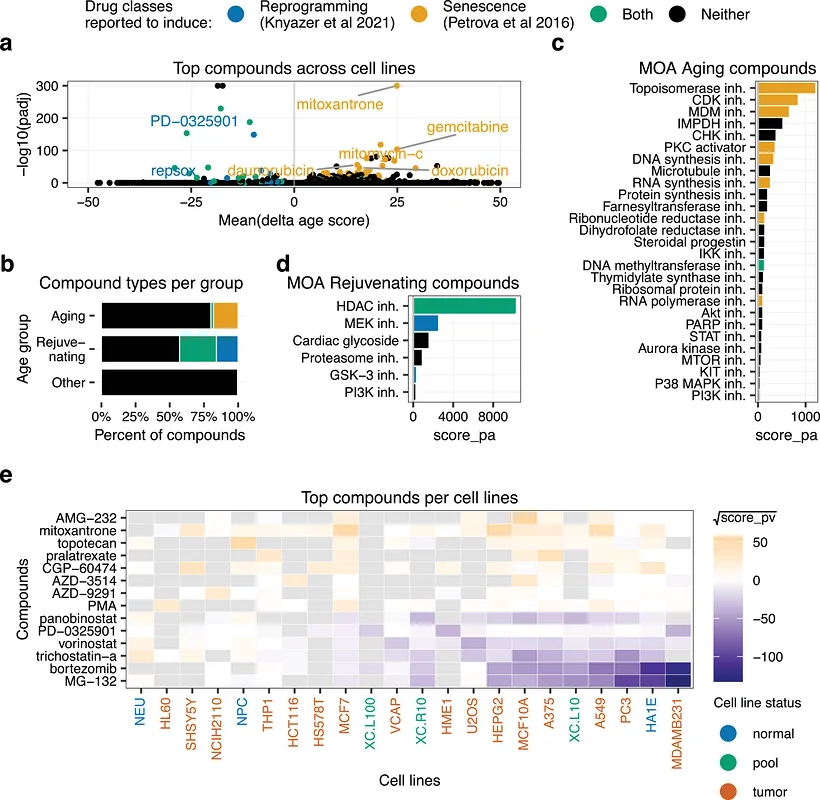
Pasta identifies age‐modulatory compounds. (a–d) Yellow, blue, green, and black indicate drug classes or compounds from drug classes that induce senescence [66], promote reprogramming [67], affect both processes, or neither, respectively. (a) Volcano plot showing the most significant Rejuvenating compounds with negative delta age scores and Aging compounds with positive delta age scores. For display purposes, p‐values equal to zero were set to 10^−300^. (b) Proportion of compounds annotated as senescence‐inducing, pro‐reprogramming, both, or neither for significantly age‐increasing (Aging), age‐decreasing (Rejuvenating), and other (Other) compounds. (c, d) Significantly enriched mechanisms of action (MOA) for Aging (c) and Rejuvenating (d) compounds. The gray lines mark the adjusted p‐value cutoff of 0.05. (e) Heatmap of compounds by cell line showing the most significant delta age scores (see Methods). Colors indicate the square root of a metric balancing significance and effect size: *‐log_10_(p‐value) × mean(delta age score)*. Missing values are shown in gray.

When repeating the analysis using the baseline regression model from Figure 1, we found the regression model to be inferior to Pasta, with the latter recovering significantly more senescence‐inducing compounds (12.7% vs 18.1%; *p* = 0.043; Fisher's exact test; Extended Data Figure S5). A similar trend was observed for the prediction of reprogramming‐promoting compounds (6.5% vs 15.9%; *p* = 0.17; Fisher's exact test; Extended Data Figure S5). Taken together, this data showed that Pasta identified age‐modulatory compounds more effectively than a conventional regression model.

Analysis of the mechanisms of action (MOAs) annotated for the CMAP compounds revealed 27 enriched MOAs among Aging compounds, including inhibition of topoisomerases, cyclin‐dependent kinases (CDKs), mouse double minute (MDMs), and inosine 5′‐monophosphate dehydrogenase (IMPDHs), consistent with DNA damage and growth arrest pathways (Figure 4c; Table S8). Six MOAs were enriched among Rejuvenating compounds (Figure 4d), dominated by HDAC inhibition, in line with its known role in accelerating reprogramming [68, 69]. Additional Rejuvenating MOAs included MAPK/ERK kinase (MEK) and glycogen synthase kinase 3 (GSK3) inhibition, common components of reprogramming cocktails [67], as well as cardiac glycosides and proteasome inhibitors. Several of these compound classes have been previously described as potential senolytics, and therefore may rejuvenate transcriptomic profiles indirectly, through selective elimination of aged cells (see Discussion).

Analyzing compound effects across cell lines revealed both conserved and context‐dependent responses. Proteasome inhibitors MG‐132 and bortezomib consistently reduced delta age scores, while mitoxantrone increased them (Figure 4e; Table S9). In contrast, several HDAC inhibitors (e.g., trichostatin‐A, vorinostat, and panobinostat) showed divergent effects, reducing delta age scores in most cell lines but increasing them in neuronal lines (NEU, NPC, SH‐SY5Y), consistent with prior reports of trichostatin‐A's cytotoxicity in SH‐SY5Y cells [73]. These results underscore the dual and context‐dependent roles of HDAC inhibitors in promoting both reprogramming and senescence [68, 69, 70, 71, 72].

Chemotherapeutics have been described as having pro‐aging properties [16, 74]. Interestingly, we observed that most approved anti‐cancer drugs were enriched in our set of Aging compounds (Note S1, Tables S10–S12, Extended Data Figure S6). Additionally, we identified compounds that had a stronger pro‐aging effect in cancer cell lines than in normal cell lines. Such candidates might have translational applications for more effective and lower toxicity anti‐cancer therapies. Furthermore, we could show that increase in cellular age is most often decoupled from increase in cell death (Note S2, Tables S13–15, Extended Data Figures S7 and S8). This indicates that applying aging clocks to perturbation datasets could lead to novel anti‐cancer therapies that have evaded conventional cell viability screens.

Collectively, these results demonstrated Pasta's ability to detect both pro‐aging and rejuvenating compounds in the CMAP L1000 data, underscoring its cross‐platform robustness and ability to reveal conserved and context‐dependent age‐regulatory interventions.

### 2.6 Experimental Validation of Compounds With Age‐Modulatory Effects

To experimentally test Pasta's predictive power in identifying cell‐type‐specific age‐modulatory compounds, we selected two top‐ranked hits, one predicted to increase and one to decrease cellular age. For each compound, two cancer cell lines were chosen: a predicted *responder* expected to show the age‐modulatory phenotype, and a *non‐responder* predicted to remain largely unaffected (Table S16).

We first examined pralatrexate, the fourth‐ranked Aging compound among 30 920 in the CMAP dataset (Table S7). In the responder A375 melanoma cells, pralatrexate induced clear signs of senescence, including cell enlargement and positivity for the Senescence‐Associated β‐Galactosidase assay (Figure 5a). It increased *CDKN1A* (p21) and *IL6* (SASP component) expression (Figure 5b), reduced proliferation as indicated by lower EdU incorporation (Figure 5c), and downregulated LMNB1 while upregulating p21 protein (Figure 5d), all consistent with a bona fide senescence response. In contrast, the non‐responder MDA‐MB‐231 breast cancer cells displayed only mild stress responses lacking key hallmarks of cellular senescence: minimal morphological changes yet detectable SA‐β‐Gal positivity (Figure 5e), negligible changes in *CDKN1A* and *IL6* mRNA levels (Figure 5f), increased proliferation and EdU incorporation (Figure 5g), and modest LMNB1 variation accompanied by upregulation of p21 protein levels (Figure 5h).

**FIGURE 5:**
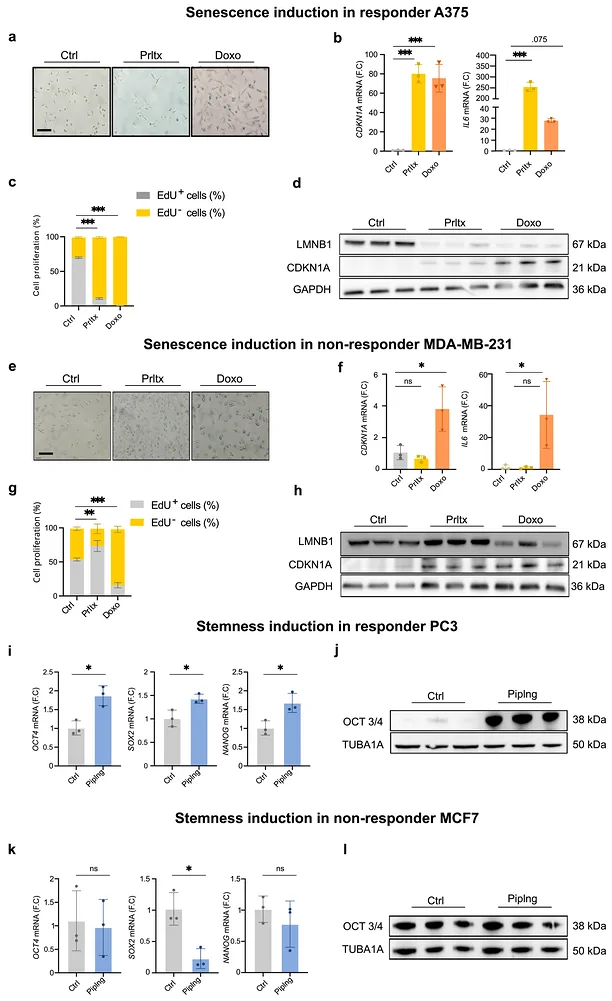
Experimental validation of pralatrexate as a senescence‐inducer and piperlongumine as a rejuvenating agent. (a–h) Testing senescence‐induction by pralatrexate in responder A375 and non‐responder MDA‐MB‐231 cells. (a, e) Representative images from Senescence Associated Beta Galactosidase assays in A375 (a) and MDA‐MB‐231 (e) cells exposed to pralatrexate. Doxorubicin served as the positive control for senescence induction. Scale bar 10 micrometers. (b, f) qPCR analysis of *CDKN1A* and *IL6* mRNA levels in A375 (b) and MDA‐MB‐231 (f) cells treated with pralatrexate or doxorubicin. N = 3 biological replicates; *p < 0.05; *** *p* < 0.001; one‐way ANOVA. (c, g) Cell proliferation by EdU incorporation in A375 (c) and MDA‐MB‐231 (g) cells after treatment with pralatrexate or doxorubicin. One representative experiment with three replicates per condition. ** *p* < 0.01; *** *p* < 0.001 (one‐way ANOVA, calculated on EdU^+^ cells). (d, h) Immunoblotting showing LMNB1, CDKN1A and GAPDH protein levels in A375 (d) and MDA‐MB‐231 (h) cells after treatment with pralatrexate or doxorubicin. N = 3 biological replicates. (i–l) Testing stemness‐induction of piperlongumine in responder PC3 and non‐responder MCF7 cells. (i, k) qPCR analysis of *OCT4*, *SOX2* and *NANOG* levels in PC3 (i) and MCF7 (k) cells exposed to piperlongumine. * *p* < 0.05; Student's t test. (j,l) Immunoblotting showing OCT3/4 and alpha‐tubulin protein levels in PC3 (j) and MCF7 (l) cells treated with piperlongumine. Abbreviations: Prltx, pralatrexate; Doxo, doxorubicin; Piplng, piperlongumine; ns, non‐significant.

Next, we evaluated Piperlongumine, ranked twentieth among 30 920 rejuvenating compounds (Table S7). In the responder PC3 prostate cancer cells, piperlongumine enhanced expression of the pluripotency factors *OCT4*, *SOX2*, and *NANOG* and increased Oct4 protein abundance (Figure 5i,j), indicating a transcriptional shift toward a youthful, stem‐like state. In contrast, non‐responder MCF7 breast cancer cells showed none of these phenotypes (Figure 5k,l).

Together, these findings confirm that Pasta accurately predicts cell‐type‐specific age‐modulatory outcomes, validating pralatrexate as a senescence‐inducing and piperlongumine as a stemness‐promoting compound.

### 2.7 Systematic Identification of Age‐Increasing and Rejuvenating Genetic Perturbations in the CMAP L1000 Dataset

To uncover genetic drivers of cellular aging, we analyzed gene knockdowns, knockouts, and overexpression profiles in the CMAP L1000 dataset. Across cell lines, 862 gene perturbations (GPs) were significantly associated with higher delta age scores (*Aging GPs*) and 58 with lower delta age scores (*Rejuvenating GPs*).

The top two Aging GPs were overexpression of *KRAS* and *BRAF* (Figure 6a; Table S17). Furthermore, overexpression of three additional genes in the mitogen activated protein kinase (MAPK) pathway (*RIT1*, *SOS1*, and *MAPK1*) were in the top 12 pro‐aging GPs. This result is consistent with the tumor‐suppressive mechanism of oncogene‐induced senescence (OIS), which is commonly triggered by hyperactivation of the MAPK pathway [75]. The third best Aging GP was knockout of *CCNA2*, consistent with a prior study reporting potent senescence induction by *CCNA2* depletion [76]. The fourth and fifth best Aging GPs were knockdowns of *ERG* and *MYC*. Interestingly, even though *ERG* and *MYC* are also oncogenes, their overexpression does not seem to trigger OIS but instead repress it [77, 78]. Accordingly, *MYC* downregulation has been shown to promote senescence [78, 79].

**FIGURE 6:**
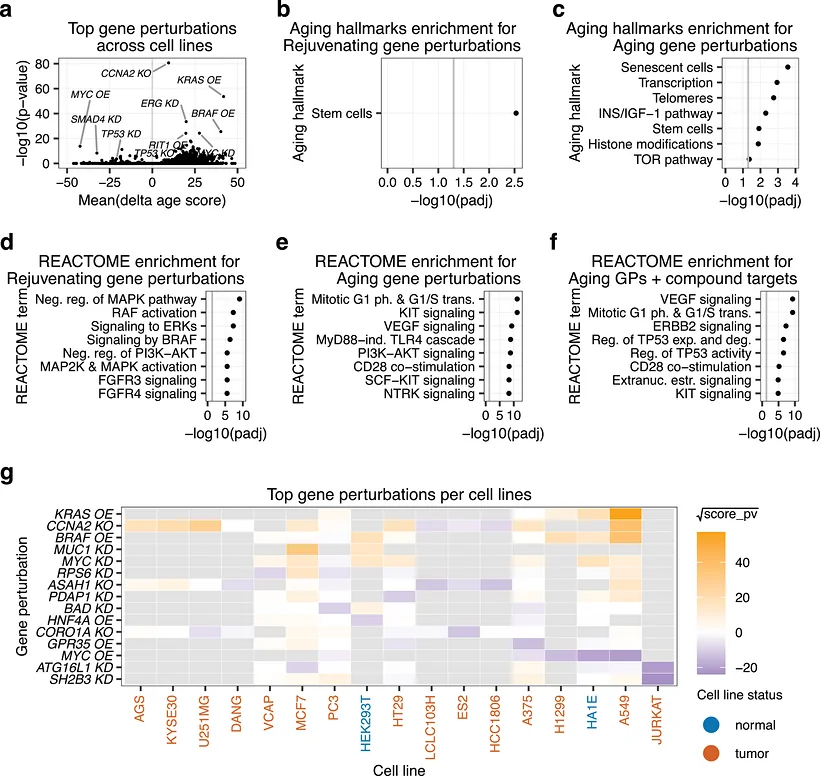
Pasta identifies age‐modulatory gene perturbations. (a) Volcano plot displaying the most significant Rejuvenating gene perturbations (GPs) with negative delta age scores and Aging GPs with positive delta age scores. (b–f) Enrichment analysis using the Hallmarks of Aging [84] (b, c) and REACTOME (d–f) gene sets for Rejuvenating pooled GPs (b, d), Aging pooled GPs (c, e), and Aging pooled GPs that are also compound targets of significant Aging compounds (f). Significant results from the three GP types (OE, KD, and KO) were pooled, with overexpression results inverted (i.e., *MYC* OE was included in the Aging GP set). Gray lines mark the adjusted p‐value cutoff of 0.05. (g) Heatmap of GPs by cell lines showing the most significant delta age scores (see Methods). Colors indicate the square root of a metric balancing significance and effect size: *‐log_10_(p‐value) × mean(delta age score)*. Missing values are shown in gray.

The top Rejuvenating GP was again connected to *MYC*—namely its overexpression—consistent with its key role in promoting stemness [80] and repressing OIS [78] (Figure 6a; Table S17). The second best Rejuvenating GP was *SH2B3* knockdown, which aligns with the reported improvement in hematopoietic stem cell function upon *Sh2b3* knockdown in *Fancd2^−/−^* mice [81]. The fourth best Rejuvenating GP was *SMAD4* knockdown, which is consistent with SMAD4's role in repressing self‐renewal of neuroblastoma stem cells [82]. Finally, *TP53* knockdown and knockout ranked as the 12th and 18th best Rejuvenating GPs. This result is consistent with the central tumor‐suppressive role of TP53 and its mutation in 50%–60% of human cancers [83].

We then systematically characterized the significant age‐modulatory GPs beyond the top hits. To this end, we inverted overexpression effects to pool all GP results and conducted gene set enrichment analysis using two complementary databases. The Aging Hallmark gene sets from the Open Genes database [84] were used to explore aging signatures, and the REACTOME database [85] gene sets were used to comprehensively study detailed cellular pathways (Figure 6b–f; Extended Data Figure S9a–f; Tables S18 and S19). Among Rejuvenating GPs, the only enriched Aging Hallmark [84] term was “stem cells” (Figure 6b), while among Aging GPs the most enriched term was “senescent cells” (Figure 6c). Aging GPs were also enriched for transcriptional regulation, insulin/IGF‐1, mTOR, and telomeres pathways, all of which are related to aging and its regulation. In REACTOME, Rejuvenating GPs were enriched in RAF/MEK/ERK and FGFR signaling (Figure 6d), whereas Aging GPs were enriched in pathways linked to cell cycle progression, receptor tyrosine kinase activation (KIT, VEGF, NTRK), PI3K/AKT signaling, and immune activation (TLR4, CD28) (Figure 6e). To get a consolidated list of age‐modulatory perturbations, we identified genes for which both genetic perturbations and compounds targeting them produced significant age effects in the same direction, yielding two Rejuvenating genes (MAP2K1 and FGFR2) and 36 Aging genes (Extended Data Figure S9g). These Aging genes were enriched in p53‐dependent cell‐cycle control and growth‐factor signaling (VEGF, ERBB2, KIT, extranuclear estrogen) (Figure 6f).

Next, we identified GPs with strong cell‐line‐specific age effects. *MYC* overexpression repeatedly produced rejuvenation, while *MYC* knockdown, and *BRAF*/*KRAS* overexpression increased cellular age (Figure 6g; Table S20). Others, such as *SH2B3* knockdown and CCNA2 and *ASAH1* knockout, could either increase or decrease cellular age depending on context, underscoring the cell‐type specificity of age regulation.

In summary, these findings map the genetic architecture of transcriptomic age modulation, revealing broad regulators like *BRAF* and *MYC* as well as context‐dependent determinants of cellular age.

### 2.8 Defining the Molecular Determinants of the Propensity of Cells to Respond to Age‐Modulatory Perturbations

While we previously revealed the pathways that drive transcriptomic age prediction (Figure 2) and that induce age‐modulation (Figures 4 and 6), it remains unclear which pathways determine the heterogeneity in responses of various cell lines to the same pro‐aging or rejuvenating cues. We addressed this question by leveraging the extensive CMAP L1000 and DepMap datasets, by identifying molecular features that govern a cell's propensity to age or rejuvenate. *Aging and rejuvenation propensities* were computed for 16 cell lines by averaging cell line‐specific delta age scores of globally significant age‐modulatory perturbations (see Methods). This analysis revealed marked heterogeneity (Figure 7a), with MDA‐MB‐231 showing the highest rejuvenation propensity (−24.3 vs. −16.8 in MCF10A), consistent with its role as a model for aggressive, triple‐negative breast cancer [86].

**FIGURE 7:**
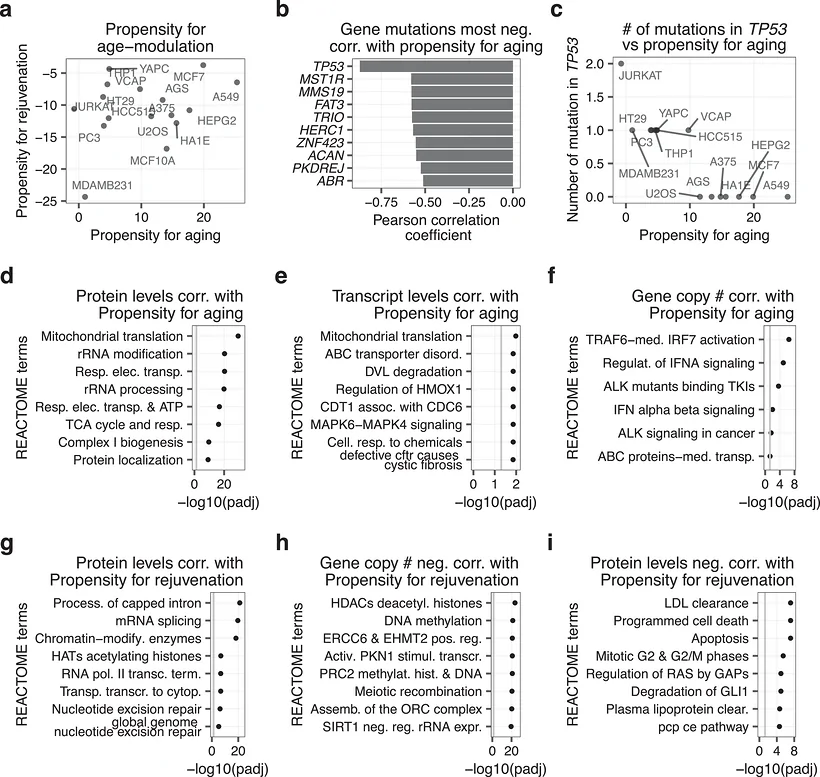
Pasta uncovers molecular determinants of cellular propensity for aging and rejuvenation. (a) Propensity of various cell lines to shift toward older or younger states following exposure to age‐regulatory perturbations (see Methods). (b) Gene mutation counts most strongly negatively correlated with the propensity for aging. (c) *TP53* mutation count versus propensity for aging. (d–i) REACTOME pathway enrichment for genes whose protein abundance (d, g, i), mRNA expression (e), and copy numbers (f, h) exhibit an absolute PCC higher than 0.4 with the propensity of cells for rejuvenation (d–f) and aging (g–i). The gray lines mark the adjusted p‐value cutoff of 0.05.

When aging propensity was correlated with the number of non‐silent mutations, *TP53* emerged as the only significantly associated gene (PCC = 0.872; Figure 7b; Table S21). Cell lines harboring two *TP53* mutations (i.e., JURKAT) had negative aging‐propensity scores, those with one mutation scored below ten, and *TP53* wild‐type lines scored higher (Figure 7c). These results align with TP53's established role as a tumor suppressor that enforces senescence in highly proliferative cells [87, 88] and suggest novel potential tumor suppressor genes (Figure 7b). Consistent with this idea, top significant genes in Figure 7b included the known tumor suppressor genes *FAT3* [89], *HERC1* [90], and *ABR* [91, 92].

To identify pathways influencing aging and rejuvenation propensities, we performed REACTOME enrichment analysis on proteins, transcripts, and gene copy numbers correlated with these traits (|PCC| > 0.4, Figure 7d–i; Extended Data Figure S10; Table S22). Proteins and transcripts associated with aging propensity were both enriched in mitochondrial translation (Figure 7d,e), underscoring mitochondrial activity as a central determinant of age‐increase. This echoes recent findings linking p53, mitochondrial translation, and the SASP (see Discussion) [93]. Copy number–based enrichments included interferon‐related pathways (Figure 7f), consistent with type I interferons' role in SASP [94], DNA damage–induced [95] and oncogene‐induced senescence [96], as well as their pro‐aging effects [97].

Rejuvenation propensity correlated with proteins involved in mRNA splicing, histone acetylation, and chromatin modification (Figure 7g). At the copy number level, HDAC, DNA methylation, and PRC2 pathways had strong negative associations (Figure 7h). These findings align with evidence that HDAC inhibition promotes reprogramming [68, 69] and that PRC2 supports pluripotency [98] and functions as a robust biomarker of aging and rejuvenation [99, 100, 101]. Proteins negatively correlated with rejuvenation propensity were enriched in apoptosis and checkpoint modules (Figure 7i). This result is consistent with the finding that elevated expression of the *Cdkn2a*/*Arf* tumor‐suppressor locus, which regulates senescence and apoptosis, constitutes a barrier for cellular reprogramming [102].

Together, these results delineate the molecular architecture of cellular age control, where mitochondrial translation and chromatin regulation act as opposing axes.

## 3 Discussion

In this study, we introduce Pasta, a multi‐tissue transcriptomic aging clock that robustly predicts relative age across diverse transcriptomic datasets. In the datasets tested in this study, Pasta achieved higher accuracy than existing transcriptomic clocks, captured cellular senescence and stemness states, and identified genetic and chemical modulators of aging. It is available as a user‐friendly, open‐source R package to support broad use in aging research.

Most published transcriptomic aging clocks are not released as usable tools [28, 32], and among the few available models, RNAAgeCalc suffers from overfitting due to training on a single dataset [29], while MultiTIMER requires extensive reprocessing and retraining for each new dataset [30], making it resource intensive, difficult to benchmark independently, and unsuitable for routine use. Moreover, both models are restricted to RNA‐Seq data, while Pasta can be used on various transcriptomic data types, including human single‐cell and mouse data, both of which were absent from the training set. Pasta achieves this through minimal preprocessing with only rank‐transformation and a novel age‐shift learning approach that compared favorably to a baseline regression model in performance, interpretability, and recovered perturbations. Finally, Pasta compared favorably with MultiTIMER [30] and the recently published multi‐species tAge clocks [33] on the human datasets examined in this study, suggesting state‐of‐the‐art performance for relative human transcriptomic age prediction.

Despite its advantages, Pasta has also some limitations. First, its relative age predictions are mainly useful for comparing age‐related effects, in years, among samples within the same dataset. When absolute age estimates are needed, the regression model, included in the pasta R package, can be used. Second, although Pasta was designed for broad use across tissues and platforms, this generality may reduce performance in uncommon conditions that are underrepresented in the training data, where a customized aging clock may be more appropriate. Related to that, while Pasta showed encouraging performance on mouse data, its predictions relied on few orthologues genes and may not be as reliable as dedicated mouse or multi‐species transcriptomic aging clocks, such as tAge. Third, applying Pasta to single‐cell transcriptomic data requires pseudobulk aggregation, which limits fine‐grained analysis of cellular states. While this is often acceptable because cells are commonly analyzed in clusters, specialized single‐cell TACs may be better suited in some settings. Fourth, Pasta was trained to predict age effects and not health‐related outcomes. While we observed association with mortality for certain cancers in TCGA, further studies will be needed to determine whether Pasta age scores are associated with specific diseases, health outcomes, or all‐cause mortality.

From a biological standpoint, Pasta captures clear links between cellular age, differentiation, senescence, and stemness. Pasta's gene coefficients and enrichment analyses converge on the DNA damage response and its downstream effector, p53 signaling, as key pathways linked to transcriptomic aging. Core predictors such as *CDKN2A*/p16 and *ZMAT3*, both canonical p53 targets, underscore this mechanistic grounding. Downstream perturbation analyses reinforced these links: topoisomerase inhibitors, which induce DNA damage, and CDK inhibitors, which block the cell cycle, were the two most enriched mechanisms of action among aging‐associated compounds, and pathways related to G1/S transition and *TP53* regulation were consistently enriched across chemical and genetic pro‐aging perturbations. Notably, recent work has proposed the DNA damage response as a fundamental “counting unit” of aging clocks, in which DDR, cell‐cycle progression, and senescence act as interconnected temporal layers of cellular timekeeping [88]. Within this framework, the transcriptomic aging patterns captured by Pasta may reflect features emerging from these distinct but coordinated biological timescales.

Pasta showed strong predictive power for identifying cellular senescence, stemness, and age‐related dynamics such as reprogramming and differentiation, despite not being trained on senescence or stem cell data. Notably, reprogramming showed ∼10‐fold stronger age effects than senescence induction (∼300 vs ∼30 years, respectively). This could reflect under‐calibration at younger ages due to limited pediatric training data but may also represent a genuine biological signal. Indeed, iPSCs and hESCs represent well‐defined cellular states at the youngest end of the biological age spectrum, whereas senescence encompasses a heterogeneous continuum with no universally accepted boundary. Furthermore, rejuvenation needs to reverse the extensive remodeling happening to cells during embryogenesis and development, while somatic aging is more linear and progressive. The detection of ∼10 times fewer rejuvenating perturbations as compared to pro‐aging perturbations in the CMAP L1000 analysis also points toward stronger age effects needed to rejuvenate cells. In any regards, these results suggest that transcriptomic aging clocks capture local cellular aging effects along a continuum from stemness to senescence. Future studies could assess whether aging clocks trained on other molecular layers, such as DNA methylation or proteomics, display similar behavior.

Application of Pasta to TCGA tumor transcriptomes revealed cancer type–specific associations with both tumor grade and overall survival after adjustment for chronological age and clinical covariates. In most cancers where the age score improved model fit, higher age scores were associated with lower tumor grade and reduced mortality. This is biologically expected: high‐grade tumors are typically enriched for stem‐like, dedifferentiated transcriptional programs that lower Pasta's age score, and senescence can act as a tumor‐suppressive barrier that restrains grade progression in certain contexts. The minority of cancers showing the opposite pattern, such as thymoma where higher age scores associated with worse survival, may reflect contexts where senescence promotes tumor progression through SASP‐driven inflammation and immune evasion, consistent with the well‐established dual role of senescence in cancer: tumor‐suppressive early but potentially tumor‐promoting in established disease. The direction of these associations thus depends on which program dominates in a given tumor context, determined by tissue of origin, driver mutation landscape, and disease stage. Together, these findings suggest that Pasta captures biologically relevant signatures beyond chronological age.

Analysis of the CMAP data showed marked enrichment of senescence‐inducing perturbations in the Aging group and of reprogramming‐ and stemness‐associated perturbations in the Rejuvenating group. Notably, Aging perturbations (271 compounds, 862 GPs) greatly outnumbered Rejuvenating ones (63 compounds, 58 GPs), consistent with a recent report [23], suggesting that diverse perturbations can accelerate aging, whereas rejuvenation requires higher precision. Perturbation effects were largely consistent across both cancer and normal (as defined in the CMAP cell annotation file) cell lines (Figures 4e, 6g, Tables S9 and S16), indicating conservation of aging and rejuvenation pathways in health and disease. Interestingly, chemotherapeutics were strongly enriched in pro‐aging compounds. These results are consistent with the view that many cancer drugs act by inducing senescence [74] and increasing biological age of both normal and cancer cells [16].

Despite considerable consistency with the literature, some of our results were less expected. For instance, proteasome inhibitors such as MG‐132 and bortezomib have been shown to transiently enhance pluripotency markers early after treatment [103] but to ultimately suppress them at later stages [104]. Given that we identified them as rejuvenating compounds argues that Pasta might particularly capture the earlier effects of perturbations, consistent with CMAP's short exposure duration (∼24 h). Furthermore, several of our top rejuvenating agents have previously been described as senolytic agents. These include proteasome inhibitors [105, 106, 107], piperlongumine [105] and the structurally related CT‐200783, cardiac glycosides [105] such as ouabain—consistent with a recent report [23]—and MEK [108] and HDAC [105, 107] inhibitors. This raises the possibility that these compounds do not intrinsically rejuvenate cells but rather eliminate aged or damaged cells from the population.

Among the chemical perturbations that we identified, pralatrexate emerged as a previously unrecognized senescence‐inducing compound. This dihydrofolate inhibitor, approved by the FDA in 2009 for relapsed or refractory peripheral T‐cell lymphoma [109], ranked fourth for senescence induction across cell lines and was validated experimentally. Its senescence‐inducing effect mirrors those of other dihydrofolate inhibitors such as methotrexate [110, 111, 112] (ranked 98th), pemetrexed [113], and pyrimethamine [114]. Notably, pralatrexate ranked first for preferentially inducing senescence in cancer cells, suggesting potential therapeutic selectivity. These results highlight the ability of transcriptomic aging clocks such as Pasta to reveal latent pharmacological properties of established drugs.

We observed heterogeneity in cell line responses to age‐modulatory perturbations, such as HDAC inhibitors having pro‐aging or pro‐rejuvenating effects depending on the cell lines. To better understand the origins of this heterogeneity, we integrated CMAP and DepMap data to identify molecular determinants of the propensity of cells to respond to age‐modulatory cues. Transcriptomic and proteomic analyses showed that pro‐senescence responses depend strongly on mitochondrial translation, consistent with links between mitochondrial metabolism, reactive oxygen species, and senescence [115]. Mitochondrial translation promotes biogenesis and reactive oxygen species production [116], driving p21‐mediated senescence [117], while p53 enhances this process to sustain cytokine output through the SASP [93]. Increased type I interferon gene copy numbers also correlated with stronger aging responses, in line with the pro‐aging role of interferon signaling [97, 118]. These findings suggest that mitochondrial translation and interferon pathways sensitize cells to senescence via oxidative and inflammatory mechanisms.

Rejuvenation was instead shaped by mRNA splicing and epigenetic factors. Reduced *PRC2* and HDAC gene copy numbers heightened sensitivity to rejuvenating stimuli, consistent with *PRC2*’s role as a universal biomarker of aging and rejuvenation [99] and with HDAC inhibition facilitating reprogramming [119]. Together, these results delineate key molecular pathways underlying propensity for cellular aging and rejuvenation and highlight potential targets for therapeutic modulation.

In summary, this study yields six main findings. First, we introduce an age shift–based transcriptomic aging clock that robustly generalizes across tissues and platforms, outperforming existing clocks on tested datasets. Second, we show that transcriptomic aging signals are dominated by a conserved DNA damage and p53‐dependent transcriptional program. Third, we demonstrate that transcriptomic age captures a continuous spectrum from pluripotency to senescence, enabling unified analysis of stemness, differentiation, and aging‐related cell states. Fourth, we establish that large scale perturbation datasets can be systematically mined to identify genetic and chemical modulators of cellular aging and rejuvenation. Fifth, we found that most age‐modulatory perturbations increase rather than decrease biological age, and that anti‐cancer agents are highly enriched in age‐increasing perturbations. Finally, we identify mitochondrial translation and chromatin‐regulatory pathways as key determinants of cellular sensitivity to aging and rejuvenation cues. Together, these findings position transcriptomic aging clocks as scalable tools for both mechanistic aging research and therapeutic discovery.

To conclude, Pasta is a biologically grounded and versatile transcriptomic aging clock. Its broad utility spans fundamental research on aging, senescence, and stem cell biology, as well as clinical diagnostics and therapeutic discovery. By enabling systematic screening of transcriptomics‐based perturbation datasets, it extends the use of conventional aging clocks toward data‐driven inference of determinants of cellular biological age. Given the rapid expansion of transcriptomics‐based perturbation datasets, this framework provides a scalable platform for translational research in cancer [16], neurodegeneration [17], regeneration [18, 19, 20], and age‐related interventions.

## 4 Methods

### 4.1 Construction of the Aging Transcriptomic Database

GTEx Protected Access Data version 8 (17,382 samples) was used to obtain precise age information [38]. We discarded 1674 samples with a RIN score below 6. An additional 1,137 samples originating from gender‐specific tissues (cervix, fallopian, uterus, prostate, ovary, testis, vagina), tissues with low sample sizes (e.g., bladder, 12 samples), or non‐relevant sources (e.g., *cells_ebv_transformed_lymphocytes*) were further discarded. Raw or normalized count matrices were retrieved in R using the package GEOquery [120] for GEO [22] datasets and using the package ExpressionAtlas [39] for Expression Atlas [121] datasets. The biomaRt R package [122] was used to convert gene or probe IDs into Ensembl gene IDs. Custom scripts were used to parse health status, age, and tissue metadata. Overall, 21 datasets with data from healthy donors were identified. These datasets contained 17 212 samples in total, including 14 571 from GTEx, and 8113 genes in common, which were also included in the landmark or best inferred gene sets in the CMAP L1000 dataset [13] and were used for further analyses. To reduce batch effect between heterogeneous datasets, a rank transformation was applied to each sample using base R's *rank* function with the parameter *ties.method = ‘average’*.

### 4.2 Generating Sample Pairs

For age‐shift models, pairs of samples from the same dataset were generated randomly and then filtered. Sample IDs were shuffled 200 times for the training set and 2000 times for the testing set within each dataset to generate IDs for both the first and the second samples in the pairs. These multipliers were chosen to ensure enough pairs are generated for both set. Then, pairs with an age difference below the cutoff (either 30, 40, or 50 years), involving non‐healthy individuals, or representing duplicate pairs (i.e., sample 1 and sample 2 swapped) were excluded. The cutoff of 40 years of age‐difference yielded the best compromise between the number of pairs to train the model and the average age effect size. Datasets with more than 1.5 times as many pairs as samples were randomly downsampled to maintain a ratio of 1.5 pairs per sample. This ratio was chosen to increase training data size while limiting overfitting. Then, the ranked gene expression values were subtracted between sample 1 and sample 2 and used as input to train the models. The output variable was binary, indicating whether sample 1 was younger or older than sample 2.

### 4.3 Training and Evaluating Models

Ridge‐regularized generalized linear models were built using 10‐fold cross‐validation to identify the optimal lambda parameters. This was done using the function *cv.glmnet* from the glmnet package [123], and using the following parameters: *s_lambda = ‘lambda.min’*, and *type.measure = ‘mse’* for regression models or *type.measure = ‘deviance*’ for classification models. Models were compared using a leave‐one‐dataset‐out (LODO) cross‐validation strategy. For classification models (traditional and age‐shift classifiers), predictions take the form of log‐odds, referred to here as age effects, obtained using the *predict* function from the glmnet package with parameter *type = ‘link’*. The Pearson correlation coefficients of the predicted ages (for the regression model) or age effects (for the classification models) with the true ages in the left‐out datasets were used as metric to compare models. Final models were then built using the same procedure, applied to all datasets, without held‐out data.

While age effects rank samples along an aging axis, they are not directly interpretable in units of years. To convert age effects into more interpretable age differences, referred to as age scores, they were multiplied by a scaling factor β specific to each classifier, defined such that the regression line of the age score on true chronological age has unit slope:

$$\begin{array}{cc} & \beta \times \mathrm{slope}\left(\mathrm{age}\;\mathrm{effect}∼\mathrm{age}\right)=1,\;\\ & \mathrm{therefore}\;\beta =1/\mathrm{slope}\left(\mathrm{age}\;\mathrm{effect}∼\mathrm{age}\right) \end{array}$$

This ensures that a one‐unit difference in age score corresponds to one year of age difference. β was estimated separately for each dataset‐tissue combination, using the age effects from that tissue in the left‐out dataset. Dataset‐tissue combinations with Pearson correlation coefficient |r| < 0.3 were excluded to ensure robustness. Per‐dataset scaling factors were calculated as the median across the remaining tissue combinations within that dataset. Finally, the classifier‐specific scaling factor was defined as the median of the per‐dataset factors. The scaling factors are included in the pasta R package and were used to obtain age scores for all analyses in the manuscript.

Because the age‐shift classifier is a linear model, the pairwise prediction f(A, B) = f(A)—f(B), where f(X) = w^T^r_X is the individual age effect of sample X, w is the learned coefficient vector, and r_X is the rank‐transformed expression vector of sample X. Consequently, the model can be applied directly to individual rank vectors to obtain age effects, and from these age scores, without requiring a paired reference sample at prediction time.

### 4.4 Analyzing Single‐Cell Transcriptomic Data

Three human aging scRNA‐seq datasets comprising lung [44], skin [46], and retina [45] transcriptomes from healthy donors were downloaded from CELLxGENE [10]. All processing steps were performed using dedicated functions from the pasta R package. Cell types with fewer than 500 cells across the dataset were removed using the filter_cell_types_in_seu_object function. Within each cell type‐donor combination, cells were randomly sampled without replacement to generate pseudobulk profiles across 11 sizes ranging from 2^0^ to 2^10^ cells, with gene expression counts summed across sampled cells, using the making_pseudobulks_from_seurat function. Pseudobulk profiles were then subset to the 8113 age‐model genes, missing genes were median‐imputed, and data were rank‐normalized using the filtering_age_model_genes_and_rank_norm function. Age scores were predicted for each pseudobulk independently, without forming sample pairs, using the predicting_age_multiple_chunks function, applying the REG, Pasta, and TC_40_60 models. Pearson correlation between true donor age and predicted age score were first computed within each cell type, then averaged across cell types for each pseudobulk size.

### 4.5 Comparing Pasta With MultiTIMER and tAge Clocks

We benchmarked Pasta against MultiTIMER [30] and six tAge [33] clocks on 14 test datasets, 8 from the LODO analysis and 6 independent datasets, using counts from ARCHS4 [9] version 2.1. LODO datasets were evaluated using Pasta's LODO predictions, while the independent datasets were assessed using predictions from the final Pasta model. Performance was assessed as the per‐dataset Pearson correlation coefficient between predicted age and true chronological age.

The MultiTIMER pipeline internally preprocesses raw ARCHS4 gene counts by applying TMM with singleton pairing normalization (edgeR) followed by log‐CPM transformation, then removes batch effects with ComBat (sva), using GEO series IDs as the batch variable. Since query and training samples must be batch corrected jointly, we re‐ran the full MultiTIMER training pipeline, pooling the 14 test datasets with MultiTIMER's 3108 training samples before normalization, batch correction, and model fitting.

The six tAge clocks combine two normalization methods (YuGene‐diff and Scaling‐diff) with three prediction targets (chronological age, normalized age, and mortality), all trained as multi‐species, multi‐tissue elastic net models. tAge models version 1 were obtained from Zenodo [124]. Preprocessing followed the tAge package (v1.0.1) pipeline: low‐count gene filtering, mapping of human gene symbols to mouse Entrez IDs via ortholog conversion, RLE normalization, log transformation, scaling or YuGene normalization, and median‐centering across all samples within each dataset.

### 4.6 Extending Pasta for Application to Mouse Samples

Orthologous gene pairs between human and mouse were obtained from the Human Gene Database HGD [125]. The table was filtered to retain only one‐to‐one orthologues. For each mouse dataset, gene symbols or probes were converted to Ensembl gene identifies. Genes were then restricted to those with one‐to‐one human orthologues and renamed using human gene names. This yielded 3292 genes, of which 1600 were among Pasta's predictive genes. Missing genes required for the Pasta age model were imputed with the dataset median, and rank‐transformations were conducted before applying the age model.

### 4.7 Analysis of the Sequencing Quality Control (SEQC) RNA‐Seq Data

The SEQC/MAQC‐III benchmark dataset (GSE47774) [47] was used to evaluate the technical reproducibility of Pasta's age scores. It comprises four reference RNA samples (A: Universal Human Reference RNA; B: Human Brain Reference RNA; C: 75% A/25% B; D: 25% A/75% B) sequenced on Illumina HiSeq 2000 at 6 independent laboratories with 4 site‐prepared library replicates per sample per site. Only site‐prepared libraries (1–4) were used for these analyses, representing a total of 96 samples. Gene‐level counts from the Bioconductor seqc package were aggregated across lanes/flow cells, converted from EntrezIDs to Ensembl gene IDs, and processed for age prediction using functions from the pasta R package. Within‐site Coefficient of Variation (CV) was calculated for each (site, sample) combination using the library replicates Pasta age scores and averaged across all combinations. Between‐site CV was calculated using site‐level mean Pasta age scores for each sample, then averaged across samples. Variance in Pasta age scores was decomposed into sample (biological), site (lab), and residual components by fitting a linear mixed‐effects model with sample and site as crossed random effects, using the lme4 R package.

### 4.8 Gene Set Enrichment Analysis

GSEA was performed using the GSEA function from the *clusterProfiler* R package [126]. Gene sets were retrieved via the msigdbr function from the *msigdbr* package [127], aggregating sets from the CGP, KEGG, REACTOME, and miRDB subcategories. Model coefficients, sorted by decreasing magnitude, were used as input. The *TERM2GENE* argument was set to the aggregated gene sets, with all other parameters left at default.

### 4.9 Analyzing Senescence Datasets

Table S1 from the SenCID manuscript [128] was used to identify senescent, proliferating, and quiescent samples. Since sample identifiers were a mix of GSM accessions, sample titles, supplementary filenames, and SRA run accessions, custom scripts resolved each to a GSM accession through four sequential lookup strategies: direct GSM matching, GEO title matching, supplementary filename matching, and SRA‐to‐GSM lookup via the NCBI SRA Accessions table. Count matrices were downloaded from GEO for 46 of 52 datasets, retaining 308 of 602 samples after ID matching and deduplication. For the replicative senescence microarray timecourse (GSE41714) [56], Illumina HumanHT‐12 V4 probe IDs were converted to Ensembl gene IDs using biomaRt (Ensembl v105). Single‐cell RNA‐Seq data from the SciPlex A549 drug screen (GSE139944) [57] were aggregated into pseudobulk profiles per drug‐dose combination using Seurat's AggregateExpression. All datasets were then preprocessed using the pasta R package following a consistent pipeline: conversion to Ensembl gene IDs (where applicable), subsetting to the 8113 age‐model genes, median imputation of missing genes, and rank normalization, before applying the Pasta age model. SciPlex‐derived age scores were matched against the Wang et al. 2017 [58] senescence‐inducing drug list using only the highest drug concentration (10 µM). AUC‐ROC values were computed using the auc function from the pROC R package [129].

### 4.10 Analyzing Stem Cell and Reprogramming Datasets

Stem cell and iPSC datasets, the OSKM reprogramming timecourse (GSE149694) [59], and the liver differentiation dataset (GSE70741) [60] were downloaded from GEO using the pasta R package function getting_GEO_ES_for_age_model, which handles EntrezID‐to‐Ensembl gene ID conversion, subsetting to the 8,113 age‐model genes, median imputation of missing genes, and rank normalization. Sample labels (ESC, iPSC, Fibroblast) were manually annotated. Age scores were predicted using pasta's function getting_pdata_with_age_scores. AUC‐ROC values were computed using the auc function from the pROC R package [129].

### 4.11 Analyzing TCGA Data—Cohort Definition and Preprocessing

Histological tumor grades were obtained from Ping et al. [130] for samples from BRCA patients and from Liu et al. [131] for samples from patients with other cancer types, as described in Zheng et al. [62]. The TCGAbiolinks R package was used to download gene expression data and clinical metadata, including age at diagnosis, survival time, and death status [132].

Only samples from primary tumors, with non‐missing values for Pasta's age score, age at diagnosis, and the relevant outcome for each analysis were kept. Within each cancer type, age score, age at diagnosis, and year of diagnosis were z‐standardized. Candidate covariates included sex, race, ethnicity, prior malignancy, standardized age at diagnosis, and standardized year of diagnosis. To avoid sparse strata, covariates were included in a cancer‐specific model only if they were estimable within that cancer type, defined as at least two levels with a minimum of 10 samples per level for categorical variables or at least two unique values for continuous variables. Only cancer types with 50 complete cases data were kept for model fitting.

### 4.12 Analyzing TCGA Data—Tumor Grade Analysis

Histological tumor grade was encoded as an ordered categorical variable with four levels (G1–G4), and cancer types with fewer than two observed grade levels after filtering were excluded from grade analyses. Associations between Pasta and tumor grade were assessed using proportional odds ordinal logistic regression. For each cancer type, a reduced model including eligible covariates and a full model additionally including standardized age scores were fitted.

### 4.13 Analyzing TCGA Data—Survival Analysis

For survival analyses, samples were required to have positive follow‐up time and cancer types with fewer than 5 death events were excluded. Overall survival was analyzed using Cox proportional hazards regression with survival time as the time variable and death status as the event indicator. Cancer‐specific reduced and full models were fitted as above.

### 4.14 Analyzing TCGA Data—Statistical Analyses

The effect of age score was reported as hazard ratios or odds ratios for higher tumor grade per 1 standard deviation increase, with 95 percent confidence intervals derived from Wald statistics. Likelihood ratio tests comparing full and reduced models were used to evaluate the incremental contribution of age score. Likelihood ratio test p‐values were adjusted across cancer types using the Benjamini–Hochberg false discovery rate procedure.

### 4.15 Analyzing CMAP L1000 Data—Preprocessing Data and Predicting Delta Age Scores

Raw level 3 data and metadata files were obtained from the https://clue.io website, and MD5 checksums were verified. Several CMAP plates had incorrect or missing annotations, which were corrected using custom scripts. Plates with missing treatment information or fewer than three control samples were excluded from all analyses. The perturbation metadata table was reduced from 3 027 596 to 3 017 946 entries after filtering. Missing mechanisms of action (MOAs) were manually annotated for 60 compounds. Missing compound names were manually annotated for seven compounds that had only BRD (Broad) IDs available. All data were split into individual .*rds* files by plate to facilitate parallel computation with reduced memory usage. For each sample, its delta age score was defined as its age score minus the mean age score of control samples on the same plate. Control samples were defined as DMSO‐treated for compound perturbations and untreated or empty vectors for genetic perturbations.

### 4.16 Analyzing CMAP L1000 Data—Annotating Senescence‐ and Reprogramming‐Related Compounds

Senescence‐inducing compounds were obtained from Petrova et al. [66]. Reprogramming‐promoting compounds were obtained from Table S1 in Knyazer et al. [67]. Due to the large number of compounds listed, only those used in at least 3 studies were selected. The selected compounds were manually checked for their presence in the CMAP dataset. In total, 31 senescence‐inducing compounds and 11 reprogramming‐promoting compounds were identified. The MOA in CMAP for each of these compounds was identified. MOAs for compounds present in both sets were annotated as ‘both’. This resulted in 5 MOAs for the ‘reprogramming’ group, 11 MOAs for the ‘senescence’ group, and 2 MOAs for the ‘both’ group. All CMAP compounds belonging to these MOAs were annotated accordingly.

### 4.17 Analyzing CMAP L1000 Data—Identification and Scoring of Age‐Modulatory Perturbations

Two‐sided t‐tests were used to assess whether delta age scores significantly differed from zero for each perturbation, either across all cell lines or within each individual cell line. All dosages, incubation times, and BRD IDs for a given perturbation were pooled to increase statistical power. Bonferroni correction was applied to account for multiple testing. For GPs, a two‐stage approach was employed to mitigate noise associated with variability in genetic construct quality. In the first stage, significance was tested for each BRD ID. In the second stage, significance was tested for each GP using only its top two BRD IDs with the highest absolute average score_pv values. Two metrics were created to combine statistical significance with effect size: score_pv = ‐log10(p‐value) * mean(delta age score), and score_pa = ‐log10(adjusted p‐value) * mean(delta age score). Perturbations were classified as Aging (score_pa ≥ −log10(0.05) and positive mean delta age score), Rejuvenating (score_pa ≥ −log10(0.05) and negative mean delta age score), or Other. This combined threshold selected perturbations that are both statistically significant (adjusted p‐value < 0.05) and had a biologically meaningful effect size (|mean delta age score| ≥ 1 year). These same definitions were applied identically for comparisons using the baseline regression model, with predicted ages substituted for age scores and the same plate‐normalization and statistical procedures applied. Over‐representation of MOAs among the significant entries, relative to other entries, was assessed using Fisher's exact test, implemented in base R's fisher.test function with *alternative = ‘greater’*. FDR correction was applied to account for multiple testing.

### 4.18 Analyzing CMAP L1000 Data—Selecting Perturbations and Cell Lines to Display in the Heatmaps

Perturbation and cell line selection was performed separately for the Aging and Rejuvenating groups. The perturbation‐by‐cell line results tables were sorted by decreasing score_pa for the Rejuvenating group and by increasing score_pa for the Aging group. The top 40 perturbations were selected for each group, with a maximum of one perturbation per cell line, which ensured that we displayed the most significant results from our study. For each selected perturbation, the top 5 cell lines were selected.

### 4.19 Analyzing CMAP L1000 Data—Pathway Enrichment Analysis

Significance results from overexpressed genes were inverted to generate a pooled set of GPs with consistent directional effects per gene. The Aging Hallmarks gene sets were obtained from Open Gene [84]. Terms were standardized to the noun forms as these were used for creating the original gene sets. REACTOME gene sets were obtained from the Human MSigDB Collections [48], using the msigdbr R package [127]. Only REACTOME pathways with 300 genes or fewer were retained (1565 of 1615). Over‐representation analysis (ORA) was performed using the *enricher* function from the clusterProfiler R package [126] with the parameter *universe* set to all the unique genes in the input database.

### 4.20 Analyzing CMAP L1000 Data—Analysis of Anticancer Drugs

Anticancer drugs were obtained from the CancerDrugs_DB [133] (build date: 28/11/24). We selected drugs approved by the FDA, the EMA, or European national agencies, which retained 311 of 321 drugs. We then kept 132 drugs that were present in the CMAP L1000 dataset. For each cancer, over‐representation of its anticancer drugs among the compounds from a given age set (Aging, Rejuvenating or Other), relative to other compounds, was assessed using Fisher's exact test, implemented in base R's fisher.test function with *alternative = ‘greater’*. FDR correction was applied to account for multiple testing.

Analysis of cancer‐specific pro‐aging compounds was conducted using one‐sided two‐sample t‐tests to assess whether mean delta age scores in tumor cell lines were greater than those in normal cell lines (as defined in the CMAP cell annotation file). P‐values were adjusted for multiple testing using FDR. Only compounds tested in at least two tumor and two normal cell lines and with a mean tumor delta age score ≥ 2 were retained. The score_pv and score_pa metrics were calculated as described above, using the difference between mean tumor and mean normal delta age scores as the effect size.

### 4.21 Analyzing CMAP L1000 Data—Identifying Consistent Compound Target/Gene Perturbation Results

To identify significant GPs whose age effects are consistent with the targets of significant compounds, we computed the mean score_pa for each gene, using the pooled GP significance table, and for each compound target. Compounds without annotated targets were excluded from this analysis. For compounds with multiple annotated targets, each target was analyzed separately. This resulted in mean score_pa values for 7,638 genes and for 867 targets of compounds. The intersection of these two sets yielded 813 entries, of which 590 had a consistent directionality of effect and were retained. 47 of these had non‐zero score_pa values in both sets and were retained. Finally, only perturbation pairs with min(score_pa) > ‐log10(0.05) were retained to include only genes significant in both genetic and chemical perturbations. This resulted in 2 genes for the rejuvenation set (*MAP2K1 and FGFR2*) and 36 genes for the Aging set.

### 4.22 Analyzing DepMap Data—Data Sources

DepMap data were obtained using the depmap R package [134]. Cell line metadata was retrieved using the *depmap_metadata* function. Drug, CRISPR, and RNAi sensitivity data were obtained using the *drug_sensitivity_21Q2*, *depmap_crispr*, and *depmap_rnai* functions, respectively. Copy number, gene expression, protein abundance, and mutation data were obtained using the *depmap_copyNumber*, *depmap_TPM*, *depmap_proteomic*, and *depmap_mutationCalls* functions, respectively.

### 4.23 Analyzing DepMap Data—Dependency Scores

The significance of delta age scores for compounds was computed for each BRD ID and cell line, using the same approach as described above (see Statistical analysis). CMAP delta age scores and DepMap dependency scores were then merged by BRD IDs for compounds (41 199 overlapping entries), and by gene names for CRISPR (46 752 overlapping entries) and RNAi (27 401 overlapping entries). The associations between dependency scores and perturbations (compound, CRISPR or RNAi) were assessed by fitting linear models with dependency score as the dependent variable and mean delta age score as the independent variable. The association between dependency scores and gene sets (MOAs for compounds and REACTOME pathways for CRISPR) was tested by fitting linear models with dependency score as the dependent variable and both mean delta age score and perturbation name as the independent variables. P‐values were adjusted for multiple testing using the Bonferroni correction. This analysis resulted in the identification of 2 MOAs, 2 compounds, 9 REACTOME pathways, and no gene perturbations whose mean delta age scores were significantly associated with dependency scores at an adjusted p‐value significance cutoff of 0.05.

### 4.24 Analyzing DepMap Data—Computing Cell Lines’ Propensity for Aging and Rejuvenation

The compound‐by‐cell line and GP‐by‐cell line delta age score significance tables were merged and filtered to retain only perturbations with significant delta age scores across all cell lines. To derive robust conclusions, the table was further filtered to retain only entries from 16 cell lines in which at least 30 significant perturbations had been tested for both the Rejuvenation and the Aging groups. Propensity scores were then computed as the mean delta age score of all significant perturbations in the Rejuvenation and Aging groups.

### 4.25 Analyzing DepMap Data—Correlating Propensity Scores With Molecular Features

The significance of the association between gene mutation and propensity scores was assessed using the Pearson product‐moment correlation coefficient test, implemented in base R's *cor.test* function. P‐values were corrected for multiple testing using the Bonferroni method. Of 1724 genes, only *TP53* showed a significant association. PCCs between cell lines’ propensity for aging or rejuvenation and protein expression levels, log‐transformed gene copy numbers, or gene expression (in transcripts per million) were computed for each molecule (e.g., gene or protein). Molecules with an absolute PCC higher than 0.4 were analyzed using ORA with the REACTOME database, as described above (see Pathway enrichment analysis).

### 4.26 Cell Culture

A‐375 (human melanoma) cells were kindly provided by Dr. Aishe Sarshad (University of Gothenburg, Sweden) and Dr. Alexander Espinosa (Karolinska Institute, Sweden). MDA‐MB‐231 (human breast cancer) cells were provided by Pr. Kirsty Spalding (Karolinska Institute, Sweden). PC3 cells were kindly provided by Dr. Yvonne Ceder (Lund University). Jurkat cells were purchased from ATCC. Cell lines were maintained in standard DMEM (Gibco). All media were supplemented with 10% heat‐inactivated fetal bovine serum (FBS; Gibco) and 1% antibiotics (penicillin/streptomycin 100 U/mL; Gibco). Cells were maintained in a humidified incubator at 37°C and 5% CO2. Cells were tested monthly for *Mycoplasma* contamination using MycoAlert Mycoplasma detection kit (Lonza LT07‐4118‐ 11630271), and only negative cells were used for experiments. For the assessment of pro‐aging or rejuvenating effects, cells were incubated in the presence of Pralatrexate (MedChemExpress, #HY‐10446, 100 nM in DMSO) or Piperlongumine (MedChemExpress, #HY‐N2329, 10 mM) for 72 h. The DNA damage‐inducing agent doxorubicin (D1515, Sigma–Aldrich, 100 nM) was used as a positive control for senescence induction.

### 4.27 SAβG Assay

Cells were washed with PBS, fixed with 0.2% glutaraldehyde for 10 min, washed with PBS, and incubated overnight at 37°C with a staining solution containing 1 mg/mL X‐Gal (Calbiochem, #203782‐1GM) prepared in dimethylformamide (DMF; Sigma–Aldrich, D4551) at pH 6. Cells were then washed in PBS and visualized using an Olympus IX73 brightfield microscope.

### 4.28 Cell Proliferation Analysis

Cell proliferation was assessed by EdU incorporation by Click‐iT Plus EDU Alexa Fluor 647 assay (C10634, Thermo Fisher Scientific) as described by the manufacturer. EdU positivity was assessed by Flow Cytometry using a BD FACSCanto II (BD) at the Biomedicum Flow Core Facility. FACS data were analyzed using the FlowJo software.

### 4.29 Immunoblotting

For immunoblotting, proteins extracted by cellular lysis in RIPA buffer were run on 4–12% Bis‐Tris acrylamide gels (#NP0322, Thermo Fisher Scientific) and electrotransferred to 0.2 µm polyvinylidene fluoride (PVDF) (#11704156, Biorad) using Trans‐Blot Turbo Transfer System (BioRad). Non‐specific binding sites were saturated by incubating membranes for 1 h in 0.05% Tween 20 (#P9416, Sigma Aldrich) v‐v in Tris‐buffered saline (TBS) supplemented with 5% BSA (#10735078001, Sigma–Aldrich, w:v in TBS), followed by an overnight incubation with primary antibodies specific for Waf1/Cip1/CDKN1A p21 (#sc‐6246, Santa Cruz Biotechnology), LaminB1 (12987‐1‐AP, Proteintech) and Oct3/4 (sc‐5279, Santa Cruz Biotechnology). Equal protein loading was monitored with GAPDH (#2118, Cell Signaling) and alpha‐tubulin (#2144, Cell Signaling) specific antibodies. Membranes were cut in order to allow simultaneous detection of different molecular weight proteins. Membranes were developed with suitable horseradish peroxidase conjugates followed by chemiluminescence‐based detection with the Cytiva Amersham ECL Prime (#RPN2232, GE Healthcare) and the Amersham ImageQuant500 software‐assisted imager (Cytiva).

### 4.30 Gene Expression Analysis by RT‐qPCR

Total RNA was extracted from cell samples using TRIzol reagent (Invitrogen, #15596018) according to the manufacturer's instructions. Up to 1 µg of total RNA was reverse transcribed into cDNA using the iScript cDNA Synthesis Kit (Bio‐Rad, #1725038). Quantitative real‐time PCR was performed using GoTaq PCR Master Mix (Promega, #A6002) in a QuantStudio 5 Real‐Time PCR System (ThermoFisher). The average expression of *ACTB* (for detection of senescence genes) or *GAPDH* (for detection of stemness genes) served as endogenous normalization controls. Primers used in this study are the following:

**Table:** 

Gene	Forward	Reverse
*CDKN1A*	CCGCCCCCTCCTCTAGCTGT	CCCCCATCATATACCCCTAACACA
*IL6*	TACCCCCAGGAGAAGATTCC	TTTTCTGCCAGTGCCTCTTT
*OCT4*	CCTGAAGCAGAAGAGGATCACC	AAAGCGGCAGATGGTCGTTTGG
*SOX2*	GCTACAGCATGATGCAGGACCA	TCTGCGAGCTGGTCATGGAGTT
*NANOG*	TTTGGAAGCTGCTGGGGAAG	GATGGGAGGAGGGGAGAGGA
*GAPDH*	AATGAAGGGGTCATTGATGG	AAGGTGAAGGTCGGAGTCAA
*ACTB*	GTTGTCGACGACGAGCG	GCACAGAGCCTCGCCTT

### 4.31 Use of Artificial Intelligence

All analyses and the initial draft of the manuscript were produced solely by the authors without the use of artificial intelligence tools. After completion of the first full draft, ChatGPT (OpenAI) was used only for language editing, including corrections to grammar, spelling, and stylistic clarity. No AI tools were used to generate data, perform analyses, or contribute to the scientific content or interpretations presented in this study.

## Author Contributions

J.S. performed all computational analyses, developed the R package, generated the computational figures and tables in the manuscript, and wrote the first draft of the manuscript. M.T., P.M.G., and E.R.D. conducted the in vitro experiments. F.P., M.T., and P.M.G. generated the figures and associated methods for the in vitro experiments. J.S., H.A., F.P., and C.G.R. edited and revised the manuscript. J.S., F.P., and C.G.R. conceived the study. F.P. and C.G.R. provided the funding.

## Funding

This work was supported by grants from the Swedish Research Council (VR; 2017–06088, 2019–04868, 2023–04383, and 2023‐02156), the Swedish Cancer Society (Cancerfonden; 20 1034 Pj and 23 2994 Pj), the Novo Nordisk Foundation (NNF21OC0070427 and NNF22OC0078353), Karolinska Institute (KID2016‐00207 and KID2021‐00495), by an ICMC project grant and by an Impetus grant to C.G.R., and by grants from the Swedish Research Council (MH 2023–02156), the Swedish Cancer Society (24 3543 Pj; 23 0690 JIA), Impetus Grants, Rosenkranz Foundation and Hevolution Foundation, Wellcome Leap's Dynamic Resilience Program (jointly funded by Temasek Trust), Ming Wai Lau Centre for Reparative Medicine (MWLC), Karolinska Institute Senior Researcher Grant, and Karolinska Institutet Blue Sky Grant for Innovative Cancer Research & Technology to Federico Pietrocola and from Wenner‐Gren Stiftelserna (UPD2024‐0048) to Federico Pietrocola and Patricia Marques Gonzalez.

## Conflicts of Interest

The authors declare no conflicts of interest.

## Supporting information




**Supporting File 1**: advs76740‐sup‐0001‐SuppMat.docx.

**Supporting File 2**: advs76740‐sup‐0002‐Extended_Data.docx.

**Supporting File 3**: advs76740‐sup‐0003‐TableS1‐S22.xlsx.

## Data Availability

All datasets used to train and evaluate the Pasta model are publicly available from the GTEx Portal (https://gtexportal.org), the Gene Expression Omnibus (GEO; https://www.ncbi.nlm.nih.gov/geo/), and the Expression Atlas (https://www.ebi.ac.uk/gxa/home). Detailed accession numbers and metadata for each dataset are listed in Table S1. The Cancer Cell Line Encyclopedia (CCLE) and DepMap datasets were accessed via the depmap R package (https://depmap.org/portal/download/). The CMAP L1000 perturbation dataset was obtained from the Connectivity Map website (https://clue.io). The open source Pasta R package, including pre‐trained models, functions for data download, processing, and age prediction, is publicly available on GitHub at https://github.com/jsalignon/pasta.
