# Supplementary material for: Pasta, a Versatile Transcriptomic Clock, Maps the Chemical and Genetic Determinants of Aging and Rejuvenation
Source: Adv Sci (Weinh). 2026 Jul 27;13(57):e76740. doi: 10.1002/advs.76740 (PMC13403736; doi:10.1002/advs.76740)
Supplement: Supplementary file 2 [file ADVS-13-e76740-s003.docx]

**Supplementary Note 1: Anticancer drugs are highly enriched in pro-aging compounds.**

Chemotherapeutic agents are widely recognized to have age-increasing- effects on both normal and malignant cells^1,2^. However, this phenomenon had not been quantified systematically. To address this, we examined cancers with at least five approved chemotherapeutics to test whether these drugs were enriched within our age-modulatory compound groups. For 17 out of 27 cancers, approved chemotherapeutics were significantly enriched in the Aging group (Extended Data Fig. 6a; Supplementary Tables 10 and 11). Only one cancer’s chemotherapeutics showed enrichment in the Rejuvenating group, while none showed enrichment in the remaining compounds. Taken together, these findings provide, to our knowledge, the first systematic quantification showing that many chemotherapeutics exert pro-aging effects^1^, and indicate the potential of transcriptomic aging clocks to reveal new candidate chemotherapeutics that increase cellular age in therapy-resistant cancer models and primary cancer cells.

By increasing cellular age, these Aging compounds drive tumor cells toward senescence, which is desirable not only because, it halts proliferation but also because it enhances tumor immunogenicity and primes the host immune system for improved surveillance^3^. A caveat to this approach is that most anticancer agents induce senescence in both malignant but also normal cells, which can reduce therapeutic efficacy, induce side-effects, and promote tumor progression or tissue remodeling^4^. The availability of both cancer and normal (as annotated in the CMAP cell annotation file) cell lines in CMAP allowed us to study this question by screening the CMAP data for compounds that induced a stronger pro-aging response in cancer than in normal cells. Pralatrexate, a dihydrofolate reductase inhibitor, showed the strongest selectivity, increasing cellular age fourfold more in cancer than in normal cell lines (Extended Data Fig. 6b-c; Supplementary Table 12). Additional promising candidates included exifone, an HDAC1 activator, and AZD−8055, an mTOR kinase inhibitor.

In summary, these results indicate that most chemotherapeutics exert pro-aging effects and that aging clocks can be used to identify candidate drugs that selectively increase the biological age of tumors which may offer a new therapeutic approach for more efficient and potential lower-toxicity cancer treatment.

**Supplementary Note 2: Screening for age-increasing compounds represents a novel strategy for identifying anti-cancer drugs and anti-cancer gene targets that could have been missed by traditional cell viability screens.**

We previously observed that most anti-cancer drugs are enriched in age-increasing compounds. This observation suggests that screening perturbation datasets for age-increasing drugs could be a novel way to identify anti-cancer drugs. However, we wondered how such a screening approach would overlap or differ from traditional viability based anti-cancer drug screening approaches. We explored this question by integrating Pasta-derived age scores with cell viability (i.e., dependency) data from the Cancer Dependency Map (DepMap)^5^ to examine how age-modulatory perturbations influence cancer cell survival.

Compounds that increased cellular age were broadly toxic to cancer cells (median dependency score -1.82, Extended Data Fig. 7a). Unexpectedly, compounds that decreased age were also toxic (median dependency score –2.94, Extended Data Fig. 7a), indicating that both pro- and anti-aging perturbations can impair proliferation, possibly reflecting broad cytostatic effects of potent compounds. To determine whether cytotoxicity levels were directly related to age-increase levels, we assessed the association between mean age scores and dependency scores for each MOA across all annotated compounds and cell lines. Only MDM and IMPDH inhibitors showed significant associations (Extended Data Fig. 7b,d; Supplementary Table 13). At the individual compound level, only AMG-232 and CGM-097, both MDM inhibitors, displayed statistically significant effects (Extended Data Fig. 7c,d; Supplementary Table 14). Therefore, few MOAs and compounds had consistent effects on age and cell viability across cell lines.

We next extended this analysis to genetic perturbations. Aging GP knockouts exhibited reduced viability (median dependency score −0.8; Extended Data Fig. 8a), while other groups showed near-zero effects. REACTOME-based analysis identified nine pathways with significant associations between age scores and dependency (Extended Data Fig. 8b; Supplementary Table 15), including retinoic acid biosynthesis (Extended Data Fig. 8c), oncogene-induced senescence (Extended Data Fig. 8d), G1/S DNA damage checkpoints, and TP53 stabilization. Therefore, again, few GP and pathways affected consistently cellular age and cell viability.

Overall, these results show that transcriptomic aging effects are largely uncoupled from cytotoxicity across both chemical and genetic perturbations. Only genetic and chemical perturbations involving pathways and genes linked to the p53–MDM2 axis, which regulates both senescence and apoptosis^6^, showed age-increases that were accompanied with increased cell death. Therefore, these findings suggest that screening for age-increasing perturbations in large scale perturbation datasets using aging clocks could lead to the identification of novel types of anti-cancer drugs and gene targets that would have been missed by traditional cell viability anti-cancer screens.

**References**

1.	Demaria, M. (2025). Cancer treatments accelerate ageing. Nat Rev Cancer, 1–2. https://doi.org/10.1038/s41568-025-00801-2.

2.	Wang, L., Lankhorst, L., and Bernards, R. (2022). Exploiting senescence for the treatment of cancer. Nat Rev Cancer *22*, 340–355. https://doi.org/10.1038/s41568-022-00450-9.

3.	Marin, I., Boix, O., Garcia-Garijo, A., Sirois, I., Caballe, A., Zarzuela, E., Ruano, I., Attolini, C.S.-O., Prats, N., López-Domínguez, J.A., et al. (2023). Cellular Senescence Is Immunogenic and Promotes Antitumor Immunity. Cancer Discov *13*, 410–431. https://doi.org/10.1158/2159-8290.CD-22-0523.

4.	Zhang, Y., Wang, T.-W., Tamatani, M., Zeng, X., Nakamura, L., Omori, S., Yamaguchi, K., Hatakeyama, S., Shimizu, E., Yamazaki, S., et al. (2025). Signaling networks in cancer stromal senescent cells establish malignant microenvironment. Proceedings of the National Academy of Sciences *122*, e2412818122. https://doi.org/10.1073/pnas.2412818122.

5.	Tsherniak, A., Vazquez, F., Montgomery, P.G., Weir, B.A., Kryukov, G., Cowley, G.S., Gill, S., Harrington, W.F., Pantel, S., Krill-Burger, J.M., et al. (2017). Defining a Cancer Dependency Map. Cell *170*, 564-576.e16. https://doi.org/10.1016/j.cell.2017.06.010.

6.	Huang, Y., Che, X., Wang, P.W., and Qu, X. (2024). p53/MDM2 signaling pathway in aging, senescence and tumorigenesis. Seminars in Cancer Biology *101*, 44–57. https://doi.org/10.1016/j.semcancer.2024.05.001.
