# Supplementary material for: Pasta, a Versatile Transcriptomic Clock, Maps the Chemical and Genetic Determinants of Aging and Rejuvenation
Source: Adv Sci (Weinh). 2026 Jul 27:e76740. Online ahead of print. doi: 10.1002/advs.76740 (PMC13403736; doi:10.1002/advs.76740)
Supplement: Supplementary file 2 — Supporting File 2: advs76740‐sup‐0002‐Extended_Data.docx. [file ADVS-9999-e76740-s001.docx]

## Supplementary Figures legends


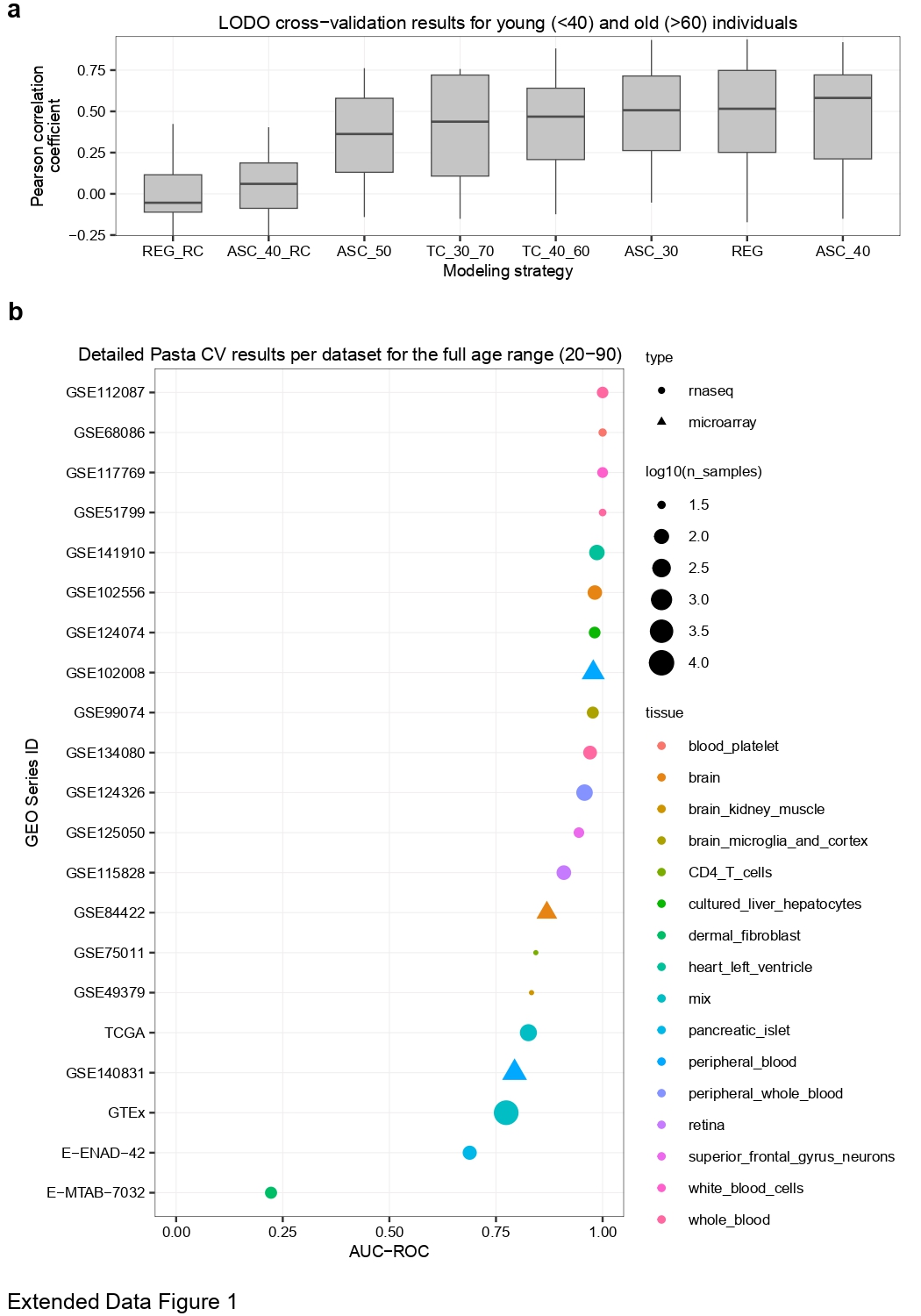


**Extended Data Fig. 1. Results of leave-one-dataset-out (LODO) analysis.**

(**a**) Comparative performance in predicting relative age, assessed with LODO, when considering only young (< 40 years old) and old (> 60 years old) individuals. (**b**) Detailed LODO results for the AS40/Pasta model when considering all individuals. The x-axis shows the AUC-ROC for predicting whether the first sample is younger than the second in each evaluation pair.


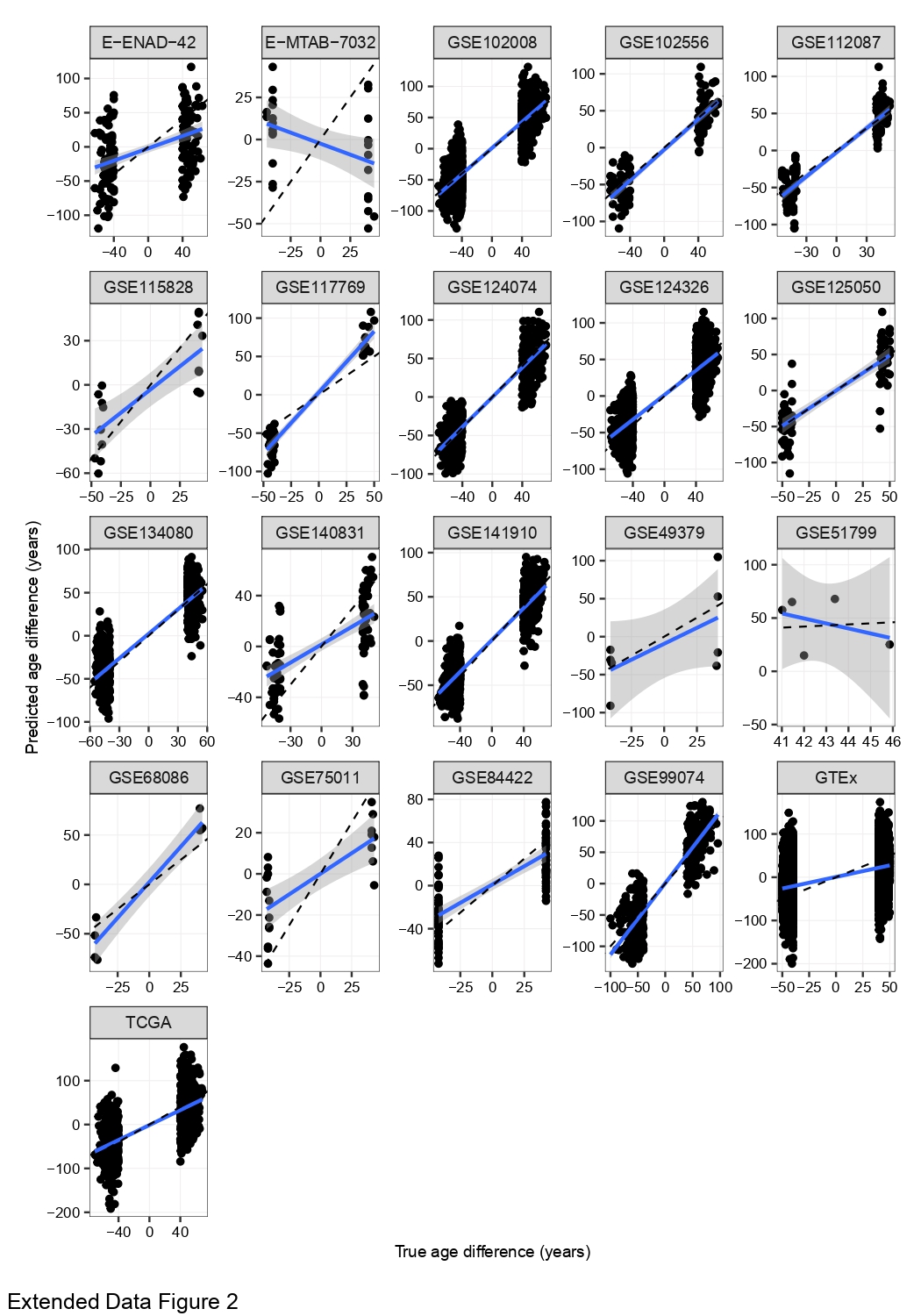


**Extended Data Fig. 2. Predicted versus true age differences for sample pairs using the AS40/Pasta LODO models.**

For each dataset, predictions were generated by a model trained on all other datasets. Each point corresponds to a sample pair with an age difference of at least 40 years.


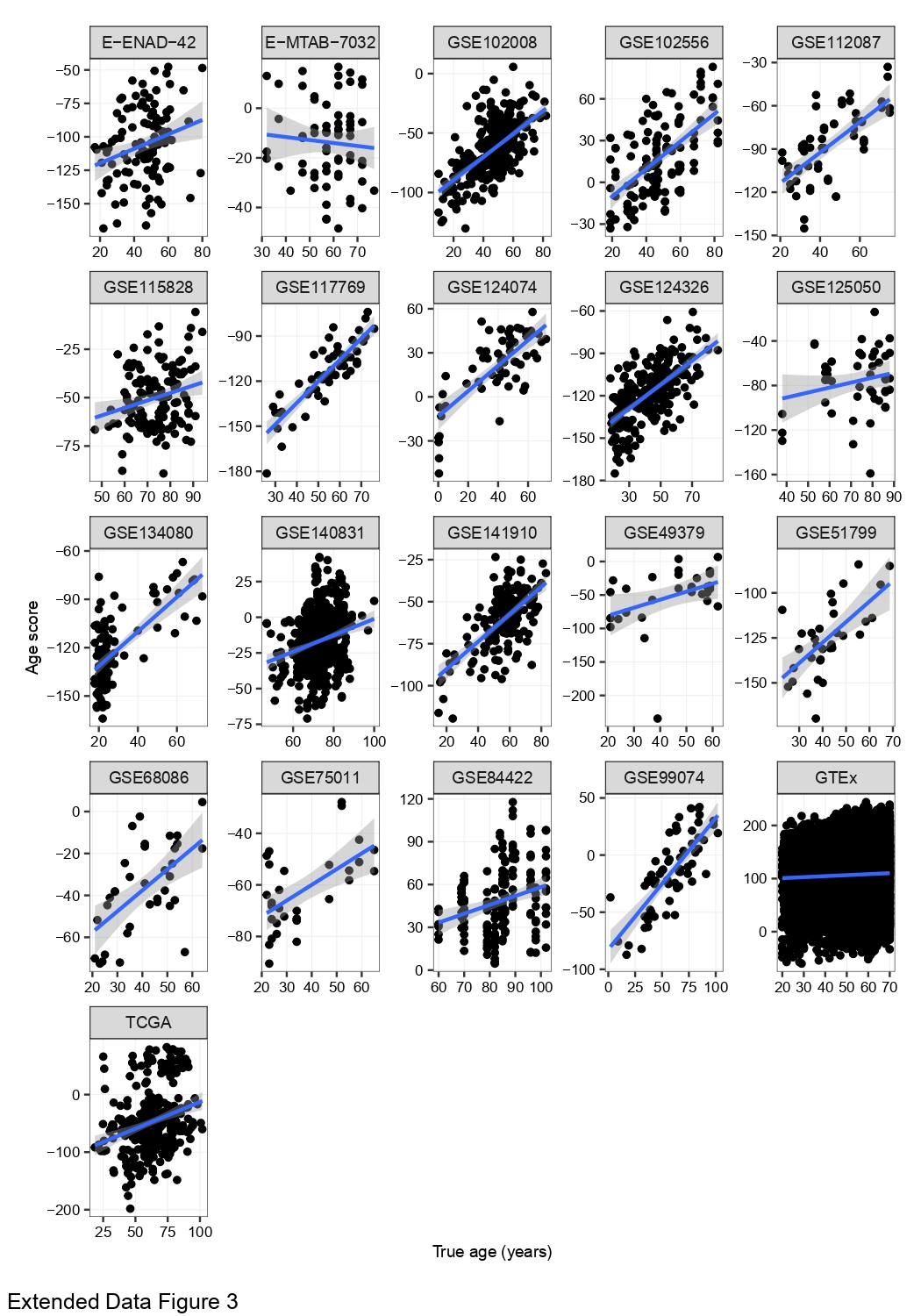


**Extended Data Fig. 3. Predicted age scores versus true ages for all samples using the AS40/Pasta LODO models.**

For each dataset, predictions were generated by a model trained on all other datasets. Each point corresponds to one sample.


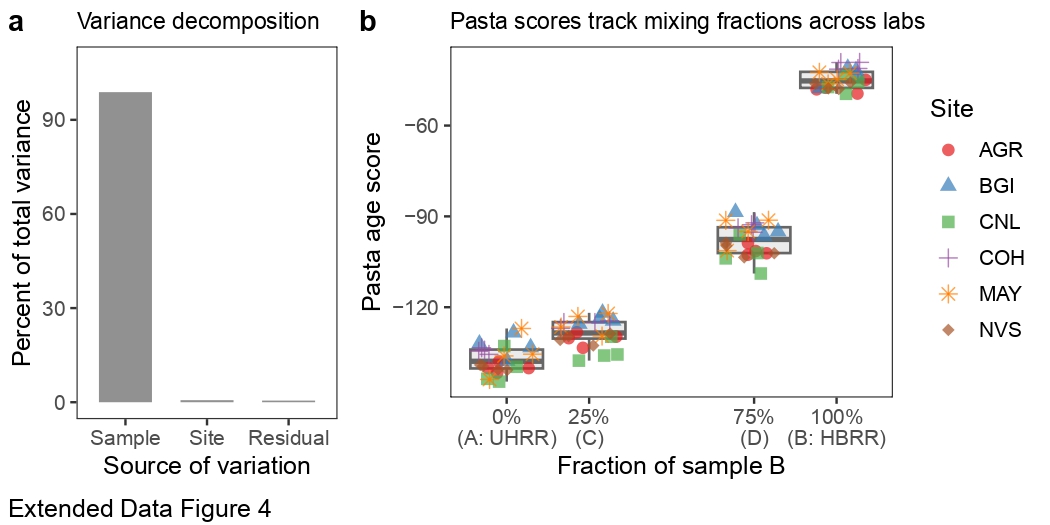


**Extended Data Fig. 4. Technical reproducibility of Pasta age scores across independent sequencing laboratories.**

1. Variance decomposition of Pasta age scores from the SEQC/MAQC-III benchmark dataset^47^, partitioned into sample (biological), site (lab), and residual components using a linear mixed-effects model with sample and site as crossed random effects. (b) Pasta age scores for four reference RNA samples sequenced at 6 independent laboratories (colored by site), plotted against their known fraction of sample B (Human Brain Reference RNA). Samples C and D are defined mixtures (25% and 75% of sample B, respectively). Boxplots show the median and interquartile range across all replicates at each mixing fraction.


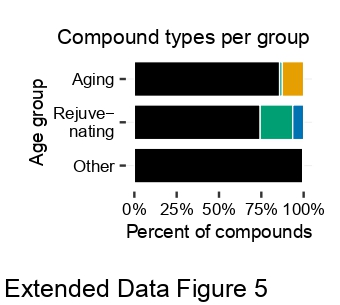


**Extended Data Fig. 5. Baseline regression model identifies fewer known age-modulatory compounds in L1000 data.**

Proportion of compounds annotated as senescence-inducing, pro-reprogramming, both, or neither for significantly age-increasing (Aging), age-decreasing (Rejuvenating) compounds, and other compounds (Other). Analyses were performed identically to Figure 4b but employed the baseline regression model from Figure 1 rather than the age-shift model.


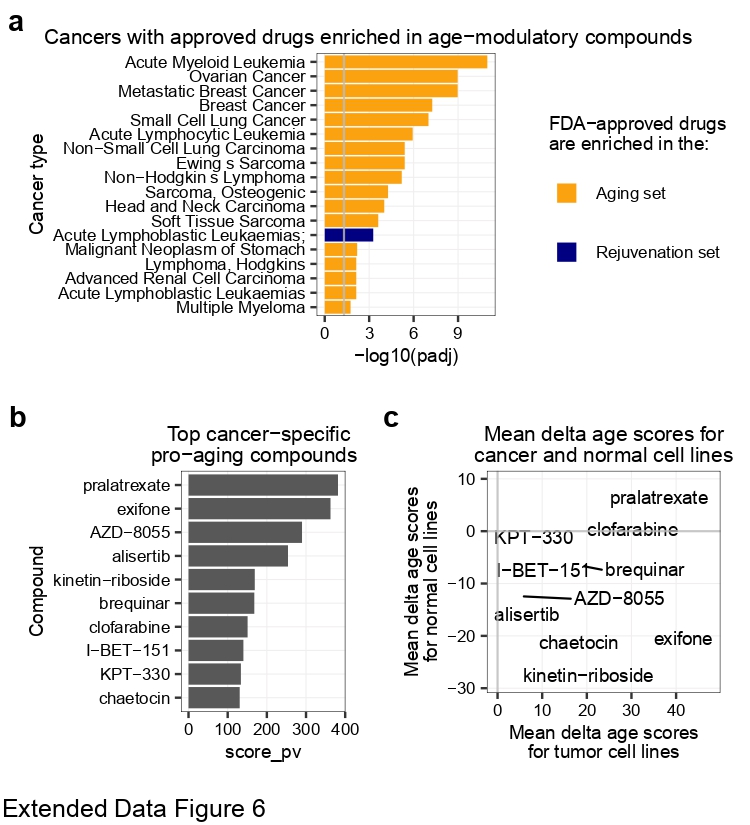


**Extended Data Fig. 6. Chemotherapeutics broadly elevate transcriptomic age.**

(**a**) Cancers whose anticancer drugs are significantly enriched in age-modulatory compounds across all cell lines. Only cancers with at least 5 approved anticancer drugs according to CancerDrugs_DB^134^ were selected. 17 of 27 cancers were significant for the Aging group. The grey line marks the adjusted p-value cutoff of 0.05. (**b**) Top 10 compounds whose delta age scores are statistically higher in cancer cell lines than in normal cell lines (see Methods). (**c**) Mean delta age score in cancer and normal cell lines for the 10 compounds shown in (b).


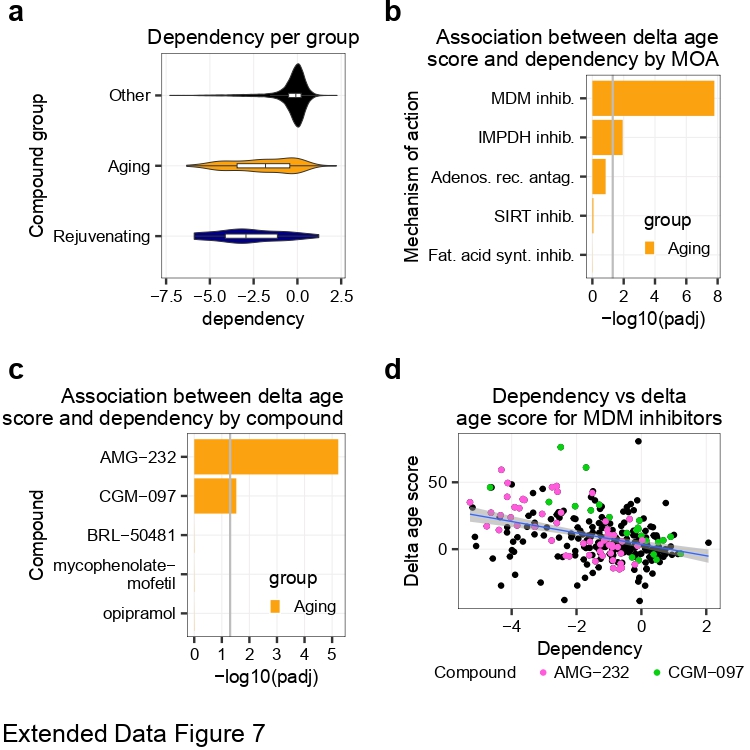


**Extended Data Fig. 7. Relationship between compound-induced changes in cellular age and viability.**

(**a-c**) Yellow, blue, and black indicate compounds in the Aging, Rejuvenating, and Other groups, respectively. (**a**) Boxplots and violin plots of dependency scores for compounds by cell line in the Aging (221 entries), Rejuvenating (76 entries), and Other (40,902 entries) groups. (**b-c**) Mechanisms of action (**b**) and compounds (**c**) with the strongest associations between delta age scores and dependency. (**d**) Mean delta age score versus dependency for MDM inhibitors. Each point represents a compound in a given cell line.


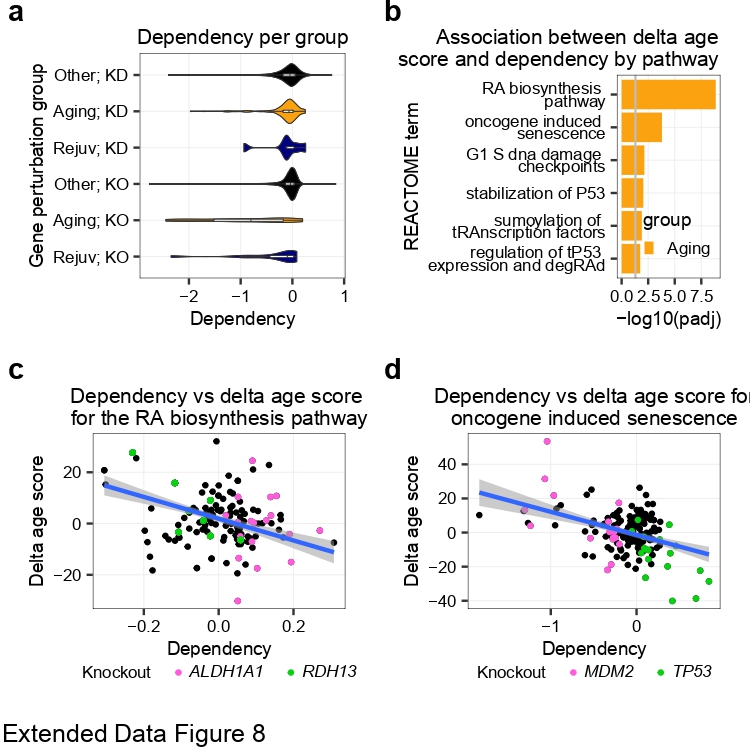


**Extended Data Fig. 8. Relationship between gene perturbation-induced changes in cellular age and viability.**

(**a-b**) Yellow, blue, and black indicate gene perturbations (GPs) in the Aging, Rejuvenating, and Other groups, respectively. (**a**) Boxplots and violin plots of dependency scores for GPs by cell line in the Aging, Rejuvenating, and Other groups. Number of entries per set: Other (KD: 27,234; KO: 46,682 entries), Aging (KD: 161; KO: 57), Rejuvenating (KD: 6; KO: 13). (**b**) REACTOME pathways whose PCCs are most significantly associated with dependency scores in the knockout Aging group. No term was enriched in the Rejuvenating group. The grey line marks the 0.05 adjusted p-value cutoff. (**c-d**) Mean delta age score versus dependency for GPs in the retinoic acid (RA) biosynthesis (**c**) and in the oncogene-induced senescence (**d**) pathways. Each point represents a gene knockout in a given cell line.


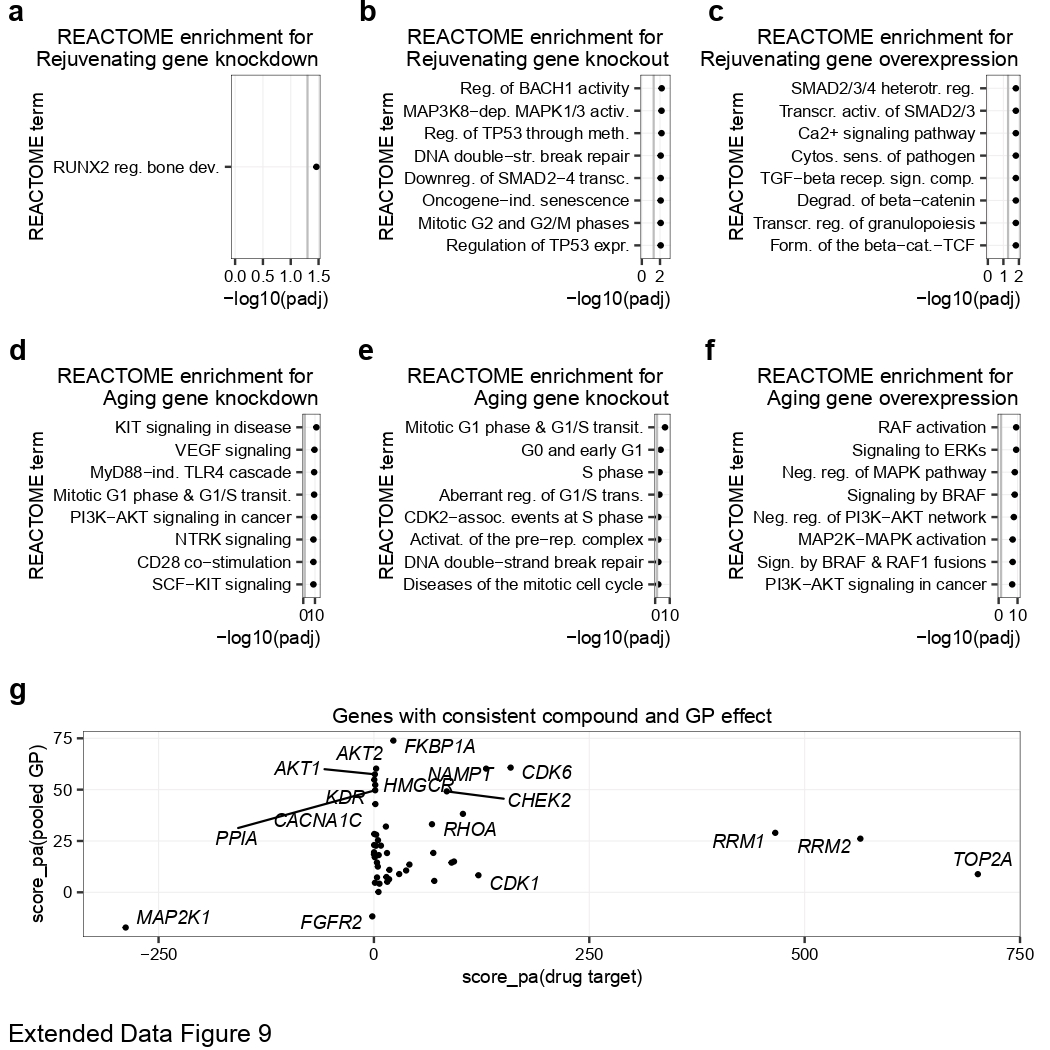


**Extended Data Fig. 9. REACTOME enrichment analysis of age-modulatory gene perturbations.**

Results for Rejuvenating knockdown (**a**), knockout (**b**), and overexpression (**c**), and for Aging knockdown (**d**), knockout (**e**), and overexpression (**f**). (**g**) Genes with significant and consistent adjusted p-value scores (score_pa) for their age effect across cell types for both the pooled gene perturbations and compound target analyses.


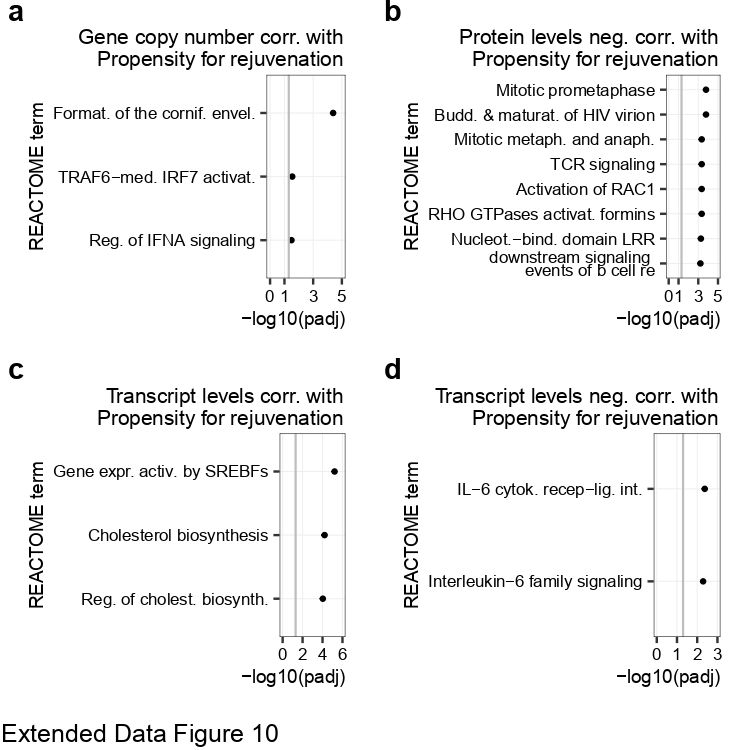


**Extended Data Fig. 10. REACTOME enrichment analysis of pathways significantly associated with cellular propensity for aging and rejuvenation.**

REACTOME pathway enrichment for genes whose copy number positively correlate with the propensity for rejuvenation (**a**), proteins whose abundance negatively correlate with the propensity for aging (**b**), and genes whose transcript levels positively correlate with the propensity for rejuvenation (**c**) or negatively correlate with the propensity for rejuvenation (**d**).
